# Emissions and Secondary Formation of Air Pollutants from Modern Heavy-Duty Trucks in Real-World Traffic—Chemical Characteristics Using On-Line Mass Spectrometry

**DOI:** 10.1021/acs.est.1c00412

**Published:** 2021-10-15

**Authors:** Liyuan Zhou, Christian M. Salvador, Michael Priestley, Mattias Hallquist, Qianyun Liu, Chak K. Chan, Åsa M. Hallquist

**Affiliations:** † School of Energy and Environment, City University of Hong Kong, Hong Kong, China; ‡ Department of Chemistry and Molecular Biology, University of Gothenburg, 412 96 Gothenburg, Sweden; § IVL Swedish Environmental Research Institute, 400 14 Gothenburg, Sweden

**Keywords:** modern vehicle emissions, roadside measurements, secondary pollutants formation, on-line chemical characterization, factor analysis

## Abstract

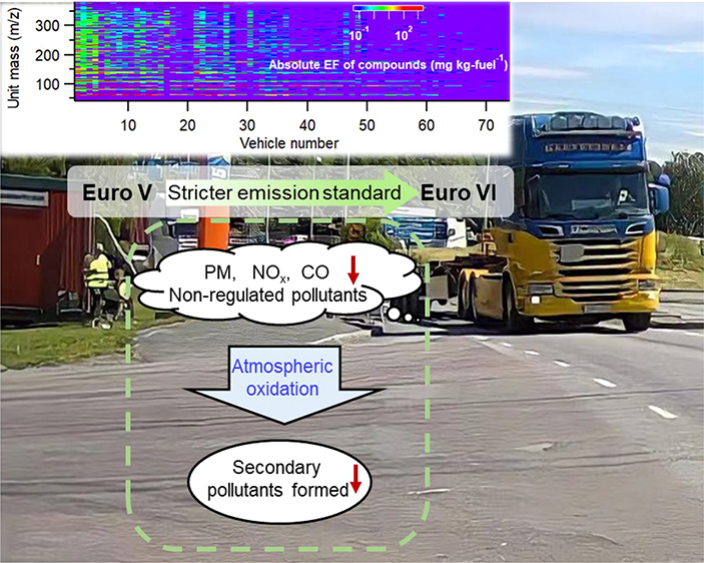

Complying with stricter emissions standards, a new generation of heavy-duty trucks (HDTs) has gradually increased its market share and now accounts for a large percentage of on-road mileage. The potential to improve air quality depends on an actual reduction in both emissions and subsequent formation of secondary pollutants. In this study, the emissions in real-world traffic from Euro VI-compliant HDTs were compared to those from older classes, represented by Euro V, using high-resolution time-of-flight chemical ionization mass spectrometry. Gas-phase primary emissions of several hundred species were observed for 70 HDTs. Furthermore, the particle phase and secondary pollutant formation (gas and particle phase) were evaluated for a number of HDTs. The reduction in primary emission factors (EFs) was evident (∼90%) and in line with a reduction of 28–97% for the typical regulated pollutants. Secondary production of most gas- and particle-phase compounds, for example, nitric acid, organic acids, and carbonyls, after photochemical aging in an oxidation flow reactor exceeded the primary emissions (EF_Aged_/EF_Fresh_ ratio ≥2). Byproducts from urea-selective catalytic reduction systems had both primary and secondary sources. A non-negative matrix factorization analysis highlighted the issue of vehicle maintenance as a remaining concern. However, the adoption of Euro VI has a significant positive effect on emissions in real-world traffic and should be considered in, for example, urban air quality assessments.

## Abstract 2

When more stringent Euro VI emission standards replaced Euro V standards, the emissions and secondary formation of both regulated and non-regulated air pollutants reduced significantly.

## 1 Introduction

Vehicular emissions are a major source of atmospheric pollutants,^1,2^ particularly in urban areas.^3,4^ In recent years, newer heavy-duty vehicles have been operated using more advanced emission control technologies, for example, diesel oxidation catalysts (DOCs) for the oxidation of CO and hydrocarbons (HC), diesel particulate filters (DPFs) to reduce particulate matter (PM), and selective catalytic reduction (SCR) systems to mitigate NO_*x*_. The changes in engine types and emission control systems and a variety of parameters, such as vehicle age, distance, and maintenance, affect vehicle emissions.^5−7^ The implementation of the Euro VI standards in Europe for heavy-duty trucks (HDTs) occurred in late 2012, and Euro VIs accounted for the highest proportion of the total distance traveled (53%) by HDTs in Sweden in 2018.^8^ Therefore, on-road measurements of the modern in-use fleet are needed to reflect real-world traffic emissions. In our recent study, we reported how this transition might have an impact on typical air pollutants such as PM, black carbon (BC), NO_*x*_, and CO. However, the emissions from HDTs do not only contain these traditional air pollutants but many other organic and inorganic compounds as well. Advanced analytical techniques, such as high-resolution time-of-flight chemical ionization mass spectrometry (HR-ToF-CIMS), can be used to further analyze emissions of these compounds from modern vehicles in real traffic.^9−11^ Furthermore, both inorganic and organic primary emissions are susceptible to secondary formation that contributes significantly to poor air quality.^1,12,13^

There is a specific lack of understanding of secondary particle formation from organic gases, that is, secondary organic aerosol (SOA),^14^ meaning that these secondary pollutants from vehicular emissions cannot be regulated directly using current regulations. Smog chambers have been used to increase the understanding of secondary aerosol production from diluted vehicle exhaust.^15−17^ Although the chamber studies can capture secondary aerosol formation from individual vehicles over several hours, they are limited to a small number of tested vehicles,^18^ which may not be representative of the actual in-use vehicle fleets. Alternatively, oxidation flow reactors (OFRs) can be used to assess secondary particle formation from vehicular emissions.^18−22^ For real-world traffic measurement studies using point sampling, OFRs with short response times are well-suited, enabling investigation of a larger number of vehicles, thus capturing the large variability between individual vehicles in a fleet.^21^ The advantage of measurements reflecting real driving conditions for a large number of vehicles is balanced by a reduced possibility to follow individuals/engines under various working conditions (e.g., engine speed and load). However, there are complementary methods that can be used for detailed characterization of various driving cycles, for example, dynamometer tests and onboard sampling. Obviously, these methods are often limited to a small sample size, and there is a challenge to accurately mimic real-world dilution. An optimum method is likely to coherently synthesize information from both approaches to extract synergistic knowledge on emission from vehicle transport systems. In this study, we performed real-world traffic measurements and focused on the in-use-trucks, where we compared the new generation of HDTs (Euro VI compliant) with slightly older models (Euro V compliant). We utilized HR-ToF-CIMS to characterize fresh and aged emissions from both gas and particle phases to reveal important differences and similarities in the transition to a modern HDT fleet. We also investigated the potential to form secondary pollutants using the OFR Gothenburg potential aerosol mass reactor, Go:PAM.

## 2 Materials and Methods

Experiments were carried out at an urban roadside site in Gothenburg, Sweden. Extractive sampling of the individual HDT plumes in real traffic was used to characterize the emission, using the method described by Hallquist et al.^23^ More details and description of the experimental conditions can be found in the paper by Zhou et al.^24^ The focus of this study was on using HR-ToF-CIMS and the OFR Go:PAM to analyze the chemical composition and the potential to form secondary pollutants. The schematic of the experimental setup and examples of temporal profiles of pollutant concentrations are shown in Figure S1 (Supporting Information). A camera was placed at the roadside to capture vehicle registration plate numbers, which were then used for further vehicle identification and to obtain engine Euro class information. The HDTs passed the sampling site at an average speed and acceleration of 33 km h^–1^ and 0.9 km h^–1^ s^–1^, respectively, on a slight uphill slope (∼2°), providing an enhanced engine load. Only plumes from Euro VI and V vehicles were included in the analysis. The criterion for a plume detection was that the CO_2_ peak concentration should exceed four times the standard deviation of the noise of the background signal. When the Go:PAM or particle characterization inlet was used, the measurements were restricted to times with lower traffic density (more time needed between plumes). Thus, the chemical characterization of the aged emission was performed on separate occasions using HR-ToF-CIMS, while the availability of two engine exhaust particle sizer spectrometer (EEPS, model 3090, TSI Inc., time resolution 10 Hz) systems enabled parallel sampling and simultaneous physical particle characterization of both fresh and aged emissions.

### 2.1 Emission Factor Measurements

A HR-ToF-CIMS system coupled with a Filter Inlet for Gases and AEROsols (FIGAERO) was used to measure both gas phase and particle phase species. A detailed description of the configuration of the instrument can be found elsewhere.^25−27^ Briefly, ambient air was sampled in the ion-molecule region (IMR) at 2.0 standard liters per minute with the species of interest being selectively ionized with reagent ions, that is, Iodide (I^–^). The data were acquired at 1 s time resolution. The post-mass calibration was fitted to a third order polynomial and for known masses (*m*/*z*) accurate within 3 ppm: NO_2_^–^, NO_3_^–^, I^–^, I·H_2_O^–^, I_2_^–^, and I_3_^–^, which covers a range of 46–381 *m*/*z*. To estimate absolute emission factors (EFs), a conversion of the CIMS signal to concentration using a sensitivity factor is necessary. Based on the method of Lopez-Hilfiker et al.,^28^ the maximum sensitivity (collision-limited) was determined to be 20 Hz ppt^–1^, which falls within previously reported ranges. Using the maximum sensitivity provides a lower-limit estimate of EFs for all the oxygenated volatile organic compounds (OVOCs).^29^ One may note that the assumption on sensitivity did not influence the observed relative emission reductions as a result of the change from Euro V to Euro VI or the ratio of aged to fresh emissions. The EFs derived using the FIGAERO were in accordance with Le Breton et al.^11^ Briefly, while conducting the gas-phase sampling/analysis, a particle sample was collected for the duration of the plume on a PTFE filter via a separate inlet. This resulted in a much shorter collection time compared to the typical ambient measurements but is compensated by a higher particle concentration in the HDT plumes. The deposited particles were directly thermally desorbed after the capturing of the plume, and the evaporated vapors were subsequently analyzed by the HR-ToF-CIMS.

The concentration of CO_2_ was measured with a nondispersive infrared gas analyzer (LI-840A, LI-COR Inc.). NO_*x*_ and NO were measured using two separate chemiluminescent analyzers (model 42i, Thermo Scientific Inc). In addition, CO, NOx, and HC were measured using a remote sensing device (AccuScanTM RSD 5000, OPUS Inspection Inc). Briefly, this instrument generates and monitors a colinear beam of IR- and UV-light passing through the plume. The pollutant concentrations are determined relative to the concentration of CO_2_ (for more details, see Hallquist et al.^23^). Vehicle speed and acceleration were also detected using the RSD. Particle emissions were measured using a high-time resolution EEPS across a size range of 5.6–560 nm. A second EEPS measured the aged particle emissions. Differences in counting efficiencies between the two EEPS were accounted for^24^ and particle wall losses in Go:PAM were corrected using size-dependent transmission efficiencies.^21^ Due to lack of detailed knowledge about the chemical composition and shape of the emitted particles, particle sphericity and unit density were assumed when calculating particle mass. BC was measured using an aethalometer at 880 nm (model AE33, Magee Scientific Inc).

The EFs of constituents per kg fuel burnt were calculated by relating the concentration change of a specific compound in the diluted exhaust plume to the change in the CO_2_ concentration compared to background concentrations^21^ to compensate for different degrees of dilution during sampling.^30^ In the calculation, complete combustion and a carbon content of 86.1% for diesel fuel were assumed (see details in Supporting Information and the paper by Zhou et al.^24^). A discussion on measurement uncertainties and variability of derived EFs is found in Supporting Information.

### 2.2 Oxidation Flow Reactor Setup

The OFR (Go:PAM) used to study the potential for the formation of secondary gaseous compounds and particle mass has been described in detail elsewhere.^21^ Briefly, the Go:PAM is a 6.1 L cylindrical continuous-flow quartz glass flow reactor with input flows such that the median residence time is approximately 37 s. The reactor is enclosed by aluminum mirrors to provide homogeneous photon fields. Two Philips TUV 30 W fluorescent lamps (λ = 254 nm) generated OH radicals through the photolysis of O_3_ (∼1000 ppb) in the presence of water vapor. The relative humidity (RH) in the reactor was 33 ± 8% (1σ) at an ambient temperature of 25 ± 2 °C. The O_3_ concentrations and RH in Go:PAM were measured continuously. The OH exposure (OH_exp_) inside the OFR was calibrated offline using SO_2_ decay, as described by Lambe et al.^31^ During the sampling, the OH_exp_ may be significantly influenced by the OH reactivity of vehicle exhaust and the titration of O_3_ with NO.^10,32,33^ Thus, the OH reactivity varies between vehicles, so it was estimated for each truck using the maximum NO_*x*_, CO, and HC concentrations in the OFR and the corresponding water and ozone concentrations (see details in Supporting Information).^21^ The use of maximum concentrations of these OH- or O_3_-consuming species represents a lower, that is, a minimum, estimate of OH_exp_ in our calculations. The estimated minimum OH_exp_ ranged from 2.0 × 10^9^ to 2.5 × 10^11^ molecules cm^–3^ s. Generally, this flow-design of an OFR enables studies of transient phenomena such as a passing plume. It also works at rather low ozone concentrations (less than 1 ppm), limiting reactions of other potential oxidants such as O_3_, NO_3_, or O^1^D.

## 3 Results and Discussion

### 3.1 Fresh Emissions

**Figure 1:**
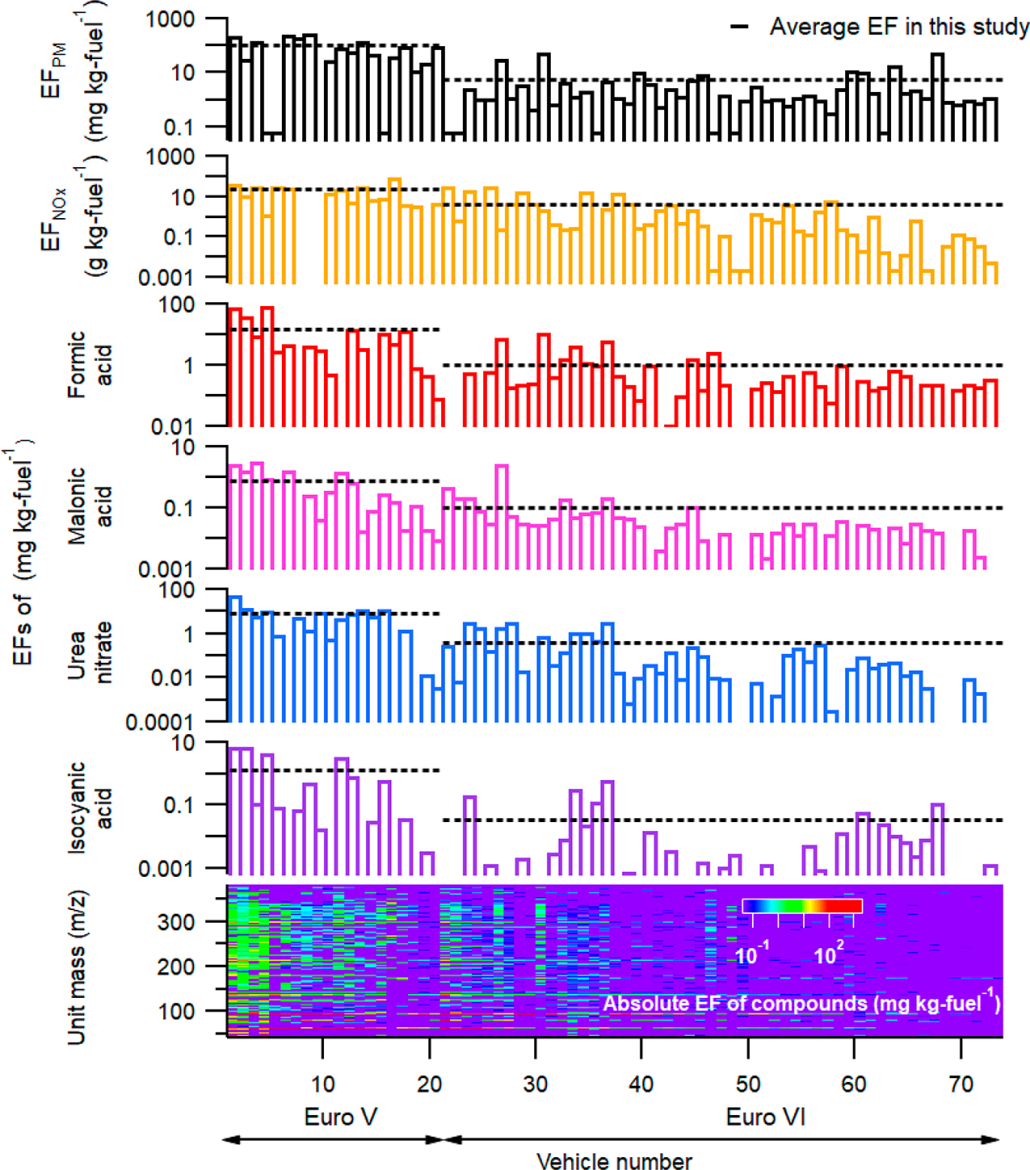
(a) EF_PM_, (b) EF_NO__x_, and EFs of gaseous (c) formic acid (CH_2_O_2_), (d) malonic acid (C_3_H_4_O_4_), (e) urea nitrate (CH_5_N_3_O_4_), and (f) isocyanic acid (HNCO) and respective average EFs (black dashed lines) and (g) EFs of compounds grouped in each unit mass for Euro V and Euro VI compliant Swedish HDTs. Non-detectable pollutant emission signals for captured plumes have been replaced with the corresponding minimum value among all recorded EFs.

Figure 1 shows the primary EFs for the particle and gas phase compounds for 18 Euro V and 52 Euro VI compliant HDTs, a subset of the total fleet of 556 HDTs described previously,^24^ and those for which HR-ToF-CIMS characterization has been carried out. Two out of the 70 HDTs passed the sampling site more than once, and their emission variabilities were small except for some conditions where there were high levels of dilution (Figure S2). For both Euro V and VI vehicles, the HDT subset had similar average EFs of, for example, particle mass (PM), particle number (PN), NO_*x*_, and CO as the full data set, being within ±35%. A thorough discussion on the full data set for fresh conditions is found in the article by Zhou et al.,^24^ while here the focus is on the results from HR-ToF-CIMS and the aged data.

In general, the EFs of general pollutants (PM, PN, and NO_*x*_) and of the majority of gaseous compounds detected by HR-ToF-CIMS were much lower for Euro VI than Euro V (Figure 1). For the lumped sum of identified non-nitrogen-containing carboxylic acids, carbonyl compounds, and nitrogen-containing organic and inorganic compounds, the average EFs were reduced by 79–94% (Table 1). A more extensive list of the changes in EFs for the identified species is shown in the Supporting Information (Table S1). The few pollutants that did not change drastically as a result of moving from Euro V to Euro VI were HONO (20% reduction), CO (40%), and PN (28%).

**Table 1:** Average EFs of Selected Gaseous Compounds Measured using HR-ToF-CIMS Complemented with General Pollutants of Euro V and VI Compliant HDTs^a^

formula	compound	Euro V (EF)	Euro VI (EF)	unit	reduction from Euro V to VI (%)
Gaseous Compounds Measured by HR-ToF-CIMS					
CH_2_O_2_	formic acid	15 ± 12 (4.2)	0.98 ± 0.60 (0.26)	mg kg fuel^–1^	93
C_3_H_6_O_3_	lactic acid	14 ± 10 (1.7)	1.1 ± 0.46 (0.42)	mg kg fuel^–1^	92
C_2_H_4_O_4_	dihydroxyacetic acid	5.5 ± 5.4^b^	0.23 ± 0.16^b^	mg kg fuel^–1^	96
C_2_H_4_O_2_	acetic acid	5.4 ± 5.5 (0.20)	0.21 ± 0.17 (0.005)	mg kg fuel^–1^	96
C_3_H_4_O_4_	malonic acid	0.73 ± 0.47 (0.23)	0.10 ± 0.10 (0.028)	mg kg fuel^–1^	86
C_3_H_6_O_2_	propionic acid	0.50 ± 0.58^b^	0.013 ± 0.011^b^	mg kg fuel^–1^	97
C_4_H_8_O_2_	butyric acid	0.91 ± 1.0^b^	0.013 ± 0.011^b^	mg kg fuel^–1^	99
C_5_H_8_O_3_	levulinic acid	3.4 ± 2.5 (0.78)	0.24 ± 0.16 (0.028)	mg kg fuel^–1^	93
C_7_H_6_O_2_	benzoic acid	2.2 ± 1.6 (0.39)	0.10 ± 0.068 (0.0072)	mg kg fuel^–1^	95
C_3_H_5_NO_5_	nitrooxypropanoic acid	1.3 ± 0.85 (0.59)	0.22 ± 0.19 (0.032)	mg kg fuel^–1^	83
C_4_H_7_NO_5_	nitrooxybutanoic acid	1.6 ± 0.76 (1.2)	0.22 ± 0.18 (0.063)	mg kg fuel^–1^	86
C_5_H_9_NO_5_	nitrooxypentanoic acid	5.1 ± 3.8 (1.5)	0.79 ± 0.93 (0.094)	mg kg fuel^–1^	85
C_6_H_11_NO_5_	nitrooxyhexanoic acid	5.0 ± 3.1 (3.3)	0.91 ± 1.1 (0.08)	mg kg fuel^–1^	82
C_6_H_5_NO_4_	dihydroxynitrobenzene	2.1 ± 1.4 (0.98)	0.13 ± 0.13 (0.022)	mg kg fuel^–1^	94
CH_5_N_3_O_4_	urea nitrate	8.2 ± 5.9 (5.6)	0.40 ± 0.25 (0.024)	mg kg fuel^–1^	95
HNCO	isocyanic acid	1.3 ± 1.2 (0.061)	0.032 ± 0.029 (0.0008)	mg kg fuel^–1^	98
HONO	nitrous acid	5.1 ± 4.2 (2.8)	4.1 ± 4.5 (0.25)	mg kg fuel^–1^	20
non-nitrogen-containing organic species (carboxylic acids and carbonyl compounds)		65 ± 56 (9.2)	4.1 ± 2.7 (0.82)	mg kg fuel^–1^	94
acidic nitrogen-containing organic and inorganic species		35 ± 26 (16)	7.4 ± 7.9 (0.59)	mg kg fuel^–1^	79
General Pollutants					
	PM	102 ± 47 (65)	5.6 ± 3.4 (1.4)	mg kg fuel^–1^	95
	PN	12.3 ± 6.8 (5.4)	8.9 ± 10.0 (0.008)	10^14^ #kg fuel^–1^	28
	BC	133 ± 104 (37)	3.6 ± 1.9 (0.23)	mg kg fuel^–1^	97
	NO_*x*_	24 ± 13 (14)	4.1 ± 2.4 (0.55)	g kg fuel^–1^	83
	CO	25 ± 9.8 (24)	15 ± 2.1 (12)	g kg fuel^–1^	40
	HC	0.47 ± 0.44^b^	0.92 ± 0.83^b^	g kg fuel^–1^	

a Stated errors are at the statistical 95% confidence interval, representing the variability of the fleet. Median EFs are shown in the parentheses. The compounds selected represent top 10 compounds regarding median EF complemented with compounds discussed in the text. A full list of all assigned compounds is given in Table S1.

b Below detection limits.

The identities of the organic compounds identified by HR-ToF-CIMS are assigned based on knowledge of sensitivities of the ionization scheme and the expected compounds emitted from the trucks. Plausible structures are assigned from the formulae, with a caveat that other isomers might contribute to the signal. The formulae listed in Table 1 have been assigned to the most plausible structures. A large proportion of the most prominent compounds identified was organic acids. The highest organic acid EFs were observed for formic acid (CH_2_O_2_) and lactic acid (C_3_H_6_O_3_). The average EF for formic acid for Euro Vs from this study (15 ± 12 mg kg fuel^–1^) falls in between that from a light-duty gasoline fleet (0.57–0.94 mg kg fuel^–1^) and ocean-going vessels (20.9 mg kg fuel^–1^)^34^ and is comparable with the EF reported in an engine dynamometer test (2.8–8.4 mg kg fuel^–1^).^10^ The average EFs for butyric acid and propanoic acid of Euro V were 0.91 and 0.50 mg kg fuel^–1^, respectively, consistent with 0.27–0.76 mg kg fuel^–1^ reported by Friedman et al.^10^ Significant reductions (up to 99%) in the average emissions of all the acids were observed for the Euro VIs in this study (see Tables 1 and S1 for EFs of more compounds).

Urea-type SCR technology is commonly used on HDTs to convert NO_*x*_ into nitrogen and water in the presence of a reducing agent, called AdBlue.^35^ We observed average EFs for urea nitrate (CH_5_N_3_O_4_) on the order of 8.2 and 0.40 mg kg fuel^–1^ for Euro Vs and VIs, respectively (Figure 1e). Urea nitrate could be formed as a reaction product of urea with nitric acid (HNO_3_). Isocyanic acid (HNCO), an intermediate by-product of thermal degradation of urea in the SCR without further sufficient hydrolysis,^36^ was detected at high concentrations in a small number of plumes (e.g., 7.8 and 7.6 mg kg fuel^–1^ for the two highest emitters in Figure 1f) possibly because it is mostly produced during the start-up/warm-up phase.^37,38^ The average EF for HNCO was 1.3 mg kg fuel^–1^ for Euro V HDTs, which is within the range of 0.21–3.96 mg kg fuel^–1^ for light-duty diesel engine emissions reported by Wentzell et al.^42^ and at the lower end of the reported values of emissions from two SCR-equipped diesel vehicles tested by Suarez-Bertoa and Astorga (1.3–9.7 mg kg fuel^–1^),^43^ while it is significantly lower than that from a non-road diesel engine with or without after-treatment systems (17–56 mg kg fuel^–1^) reported by Jathar et al.^44^ and Link et al.^45^

**Figure 2:**
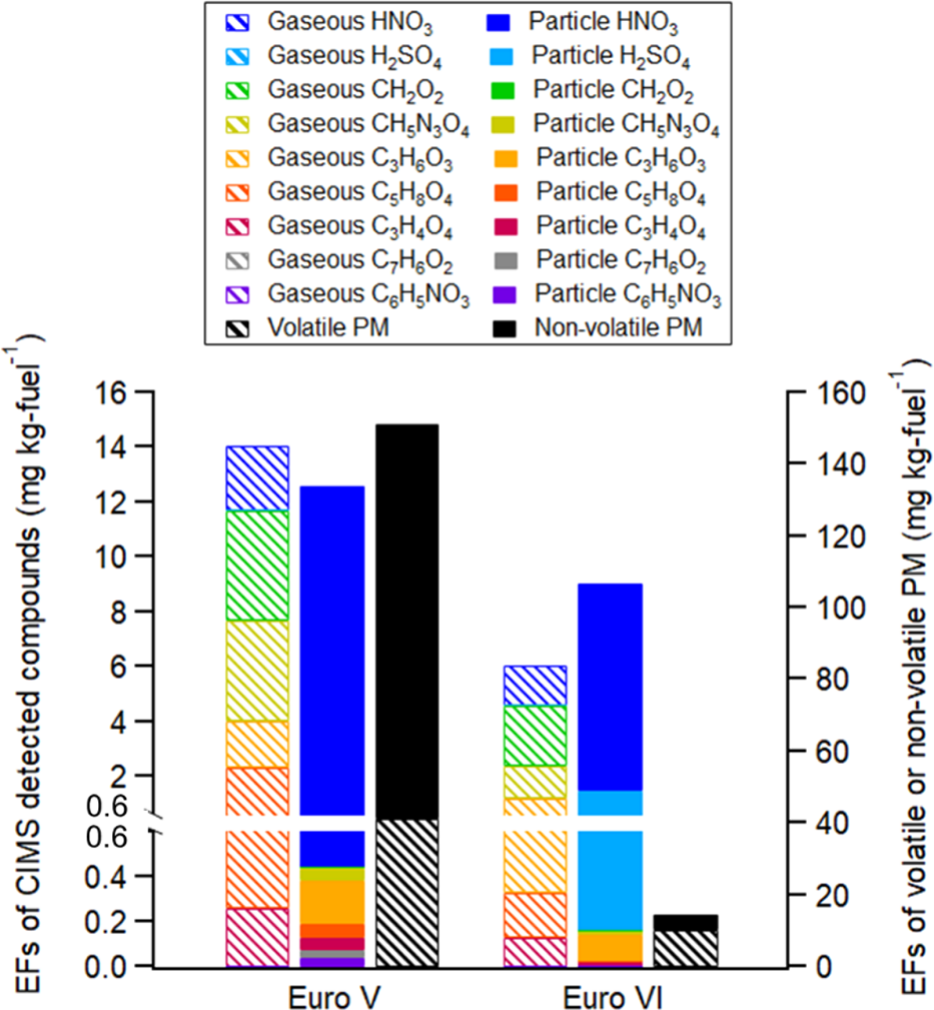
Emissions of gas- and particle-phase CIMS detected compounds, volatile-PM, and non-volatile PM for a Euro V and a Euro VI HDT.

Figure 2 shows the particle-phase pollutant EFs characterized by the FIGAERO ToF-CIMS together with the corresponding gas-phase EFs and the EFs of volatile and non-volatile (thermodenuder-conditioned) PMs for a Euro V and a Euro VI HDT (see details on thermodenuder operation and conditions in the article by Zhou et al.^24^). This provides a first insight into how the particle-phase composition may change as a result of a full transition from older (Euro V) to newer technology (Euro VI). HNO_3_ is the most abundant species detected in the particle phase, with an EF of 12.2 and 7.5 mg kg fuel^–1^ for the Euro V and VI in Figure 2, respectively, considerably higher than 2.5 mg kg fuel^–1^ for diesel-fueled buses reported by Le Breton et al.^11^ The slightly reduced HNO_3_ emissions shown in Figure 2 may be related to the small decrease in NO_2_ emissions for these HDTs (3.5 and 1.2 g kg fuel^–1^ for Euro V and Euro VI respectively), while total NO_*x*_ emissions for these decreased more significantly from 39 to 4.8 g kg fuel^–1^. The Euro VI shown in Figure 2 emits 37–68% less of the individual pollutants measured in the particle phase, than the Euro V. This is similar to the reduction in the emissions of most gaseous species. The reduction of the identified particle-phase species does not correspond to the overall decrease in total PM (90%) for these two HDTs (Figure 2). The largest reduction is due to the non-volatile fraction (96%), that is, primarily the soot fraction, which was presumed to be removed efficiently by the DPF. The volatile fraction decreased less drastically (75%) but still does not match the more modest decrease observed for the characterized species. The selectivity of the iodide ionization scheme may explain the observations where species with low volatility, such as long chain hydrocarbons, cannot be effectively measured. In general, the total EF_CIMS_ accounted for a relatively high fraction (88%) of EF_volatile-PM_ for the Euro VI but only 30% of EF_volatile-PM_ for the Euro V.

### 3.2 Non-negative Matrix Factorization and Hierarchical Clustering Analysis for Fresh Gaseous Emissions

**Figure 3:**
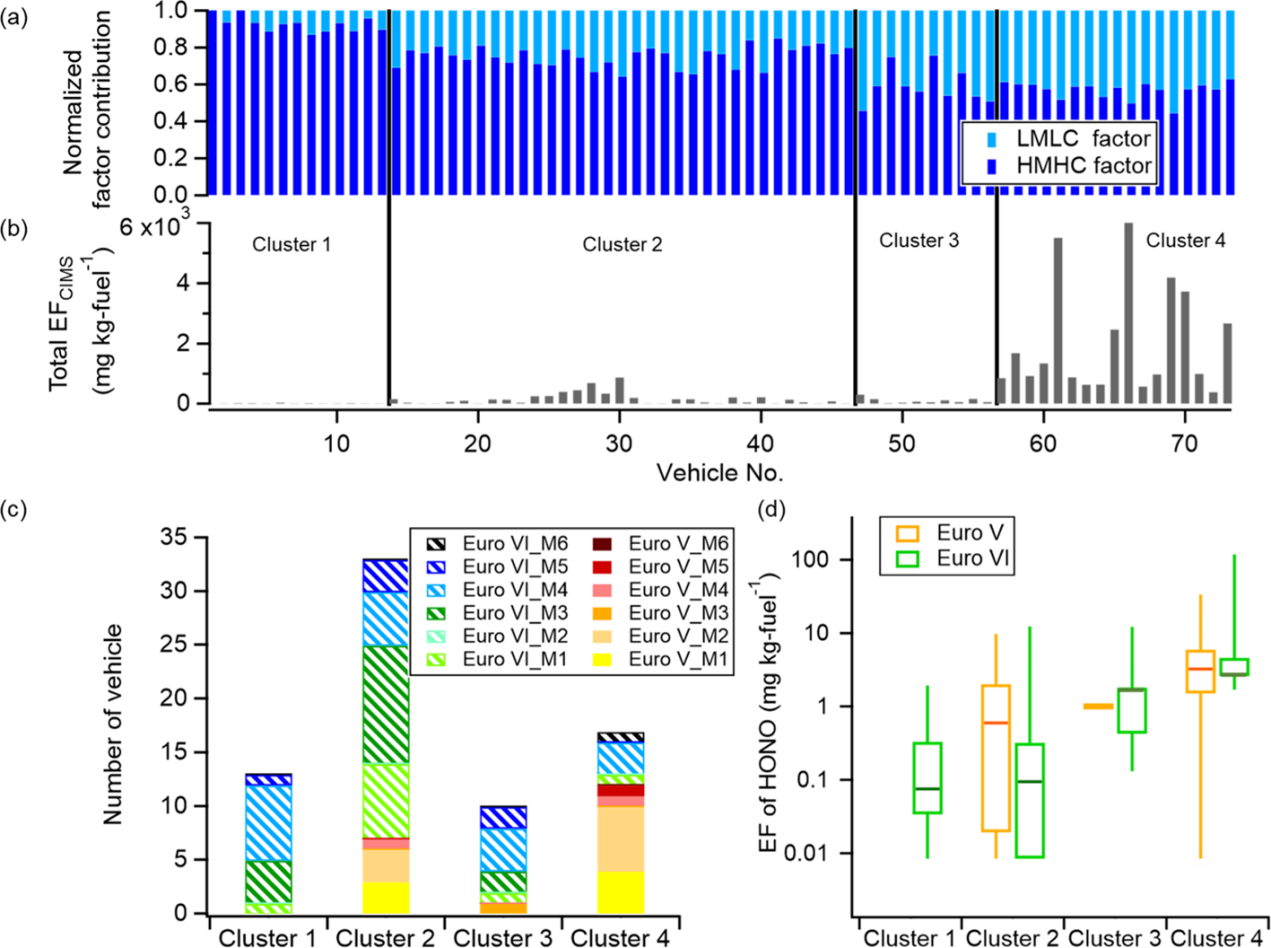
(a) Normalized contribution of HMHC and LMLC factors resolved from the NMF model, (b) EFs of groups of compounds detected by HR-ToF-CIMS, (c) HDTs from different manufacturers clustered by HCA, and (d) EFs of HONO for Swedish Euro V and Euro VI compliant HDTs. Non-detectable pollutant emission signals for captured plumes have been replaced with the corresponding minimum value of all recorded emission factors. For box and whisker plots, the top and the bottom line of the box are 75 and 25 percentiles of the data, respectively, the dark orange and green lines are the medians, and the top and bottom whiskers are 90 and 10 percentiles, respectively.

More information about the HDT emissions was obtained by analyzing the huge number of ions measured by HR-ToF-CIMS. Here, non-negative matrix factorization (NMF) together with hierarchical cluster analysis (HCA) was used to resolve the EFs of the groups of gaseous compounds from the HR-ToF-CIMS spectra (see Supporting Information). The particle phase and aged emissions were excluded because the low sampling number would cause a bias in the evaluation. The analysis was restricted to the 223 ions that had a data coverage of at least 75% in the 73 passages. From the NMF, two factors were found and designated as a high mass and high carbon (HMHC) factor and a low mass and low carbon (LMLC) factor, with average molecular mass for the top 10 contributing ions of 287 ± 62 (1σ) and 93 ± 33 Th, respectively (Figures S5, S6 and Table S2). The vehicle data were clustered by HCA using these two NMF-derived factors as input variables. A four-cluster solution was chosen as it was the simplest yet the most interpretable configuration (Figure S7), describing a mixture of the magnitude of EFs and the fraction of each factor within the clusters (Figure 3a–c). M1–6 represents the six manufacturers of the HDTs. Cluster 1 is characterized by the lowest proportion of the LMLC factor (7.4% on average) and the lowest total EF_CIMS_ and considered as the “cleanest” cluster containing only Euro VI vehicles. Cluster 2 contains a slightly higher proportion of the LMLC factor (25%) and the largest number of Euro VIs but also some EuroVs. Cluster 3 contains further low total EF_CIMS_ but shows an increasing proportion of the LMLC factor (41% on average). Cluster 4 contains mainly Euro Vs, the highest total EF_CIMS_, and a similar proportion of the LMLC factor. One possible reason for the pattern of factor distributions and total EF_CIMS_ among clusters for vehicles of the same Euro class may be related to the performance of the SCR. Liu et al.^46^ described how a SCR system influences NO_*x*_ and low molecular weight organic emissions. HONO was one of the most common contributors to the LMLC factor. The median EF_HONO_ for Euro VIs in clusters 1 and 2 was more than 90% lower than in cluster 3 (Figure 3d). A similar trend is also present for NO_*x*_ emissions (Figure S8). Increased proportions of the LMLC factor may indicate SCR deterioration, which is likely to worsen upon further distance traveled. Here, the median distances traveled by Euro VIs in clusters 3 and 4 are more than those in clusters 1 and 2 (Figure S8). To meet more stringent regulations, multiple after-treatment systems (SCR + DPF) are incorporated in Euro VIs,^47^ which may make it more difficult to maintain their optimal operating conditions in real-world traffic. However, no deterioration of DPFs is apparent because the EF_PM_ for Euro VIs is similar across different clusters (Mood’s median test, *p* > 0.05) (Figure S8). Euro VIs and Vs cannot be completely separated using NMF analysis likely because of the influence of vehicle deterioration. Therefore, a concern that remains is the issue of maintenance that will become more important in the future when emissions of pollutants during normal operation have decreased significantly.

### 3.3 Aged Emissions

In total, the aged emissions of 94 HDTs were investigated. The exhaust was oxidized in Go:PAM, and chemical characterization of the aged gaseous pollutants was carried out for 11 HDTs using HR-ToF-CIMS. As with the fresh gaseous emissions, organic acids accounted for a large proportion of the most prominent identified compounds in the aged gaseous emissions, dominated by formic acid (CH_2_O_2_) for most of the HDTs (average-aged EFs of formic acid = 4.2 mg kg fuel^–1^ for Euro VIs and 74 mg kg fuel^–1^ for a Euro V). For Euro VIs, five out of the six compounds with the highest average-aged EF were carboxylic acids, of which malonic (C_3_H_4_O_4_), lactic (C_3_H_6_O_3_), pyruvic (C_3_H_4_O_3_), and malic acid (C_4_H_6_O_5_) were the most dominant. For aged Euro V emissions, levulinic (C_5_H_8_O_3_), acetic (C_2_H_4_O_2_), pyruvic (C_3_H_4_O_3_), and lactic acid (C_3_H_6_O_3_) were the most abundant. The EFs of the top six compounds were generally a factor of ∼20 lower for Euro VIs compared to Euro Vs. Also, some nitrogen-containing organic compounds, such as nitrooxybutanoic acid (C_4_H_7_NO_5_) and nitroacetic acid (C_2_H_3_NO_4_) were observed among the compounds with the highest-aged EFs (Table S1).

**Figure 4:**
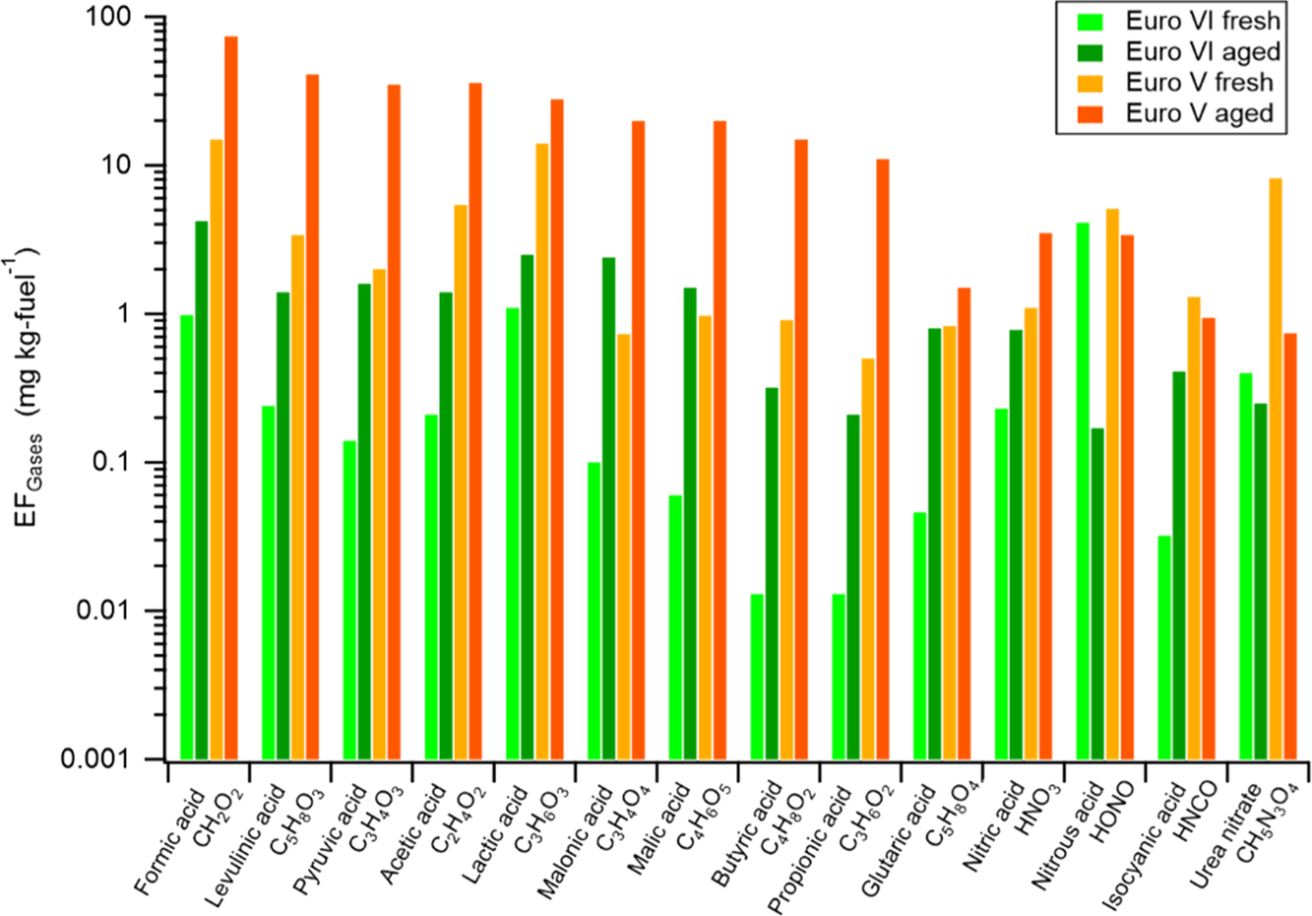
Average fresh and aged gaseous emissions for Euro VI- and V-compliant HDTs.

The average-aged gaseous emissions of carboxylic, nitric, and isocyanic acid ranged from two to more than 100 times those of the fresh emissions. However, the emissions of HONO and urea nitrate decreased by 32–96% after aging (Figure 4). The largest enhancement of aged to fresh emissions of carboxylic acids was observed for malic followed by butyric and malonic acid for Euro VIs. Malonic, propionic, and malic acids yielded the largest enhancements for Euro Vs. The secondary production of formic, butyric, and propanoic acid to similar levels has also been observed in previous laboratory chassis dynamometer engine (fueled with diesel) emission tests (0.32–32.1 mg kg fuel^–1^).^10^ Apparently, the photochemical aging of vehicle emissions enhances the importance of small acids originating from heavy-duty traffic.

Upon oxidation, secondary particle mass can be formed. The largest part of the identified secondary PM for Euro VI was due to nitric and sulfuric acid, but carboxylic acids were also significantly present. Three out of five of the most prominent identified compounds were organic acids, which is in line with the aged diesel bus emissions described by Le Breton et al.,^11^ and the aged EFs of particle-phase lactic, malonic, and nitric acid were 1–2 orders of magnitude higher than in the fresh emissions (Table S3 and Figure S9).

**Figure 5:**
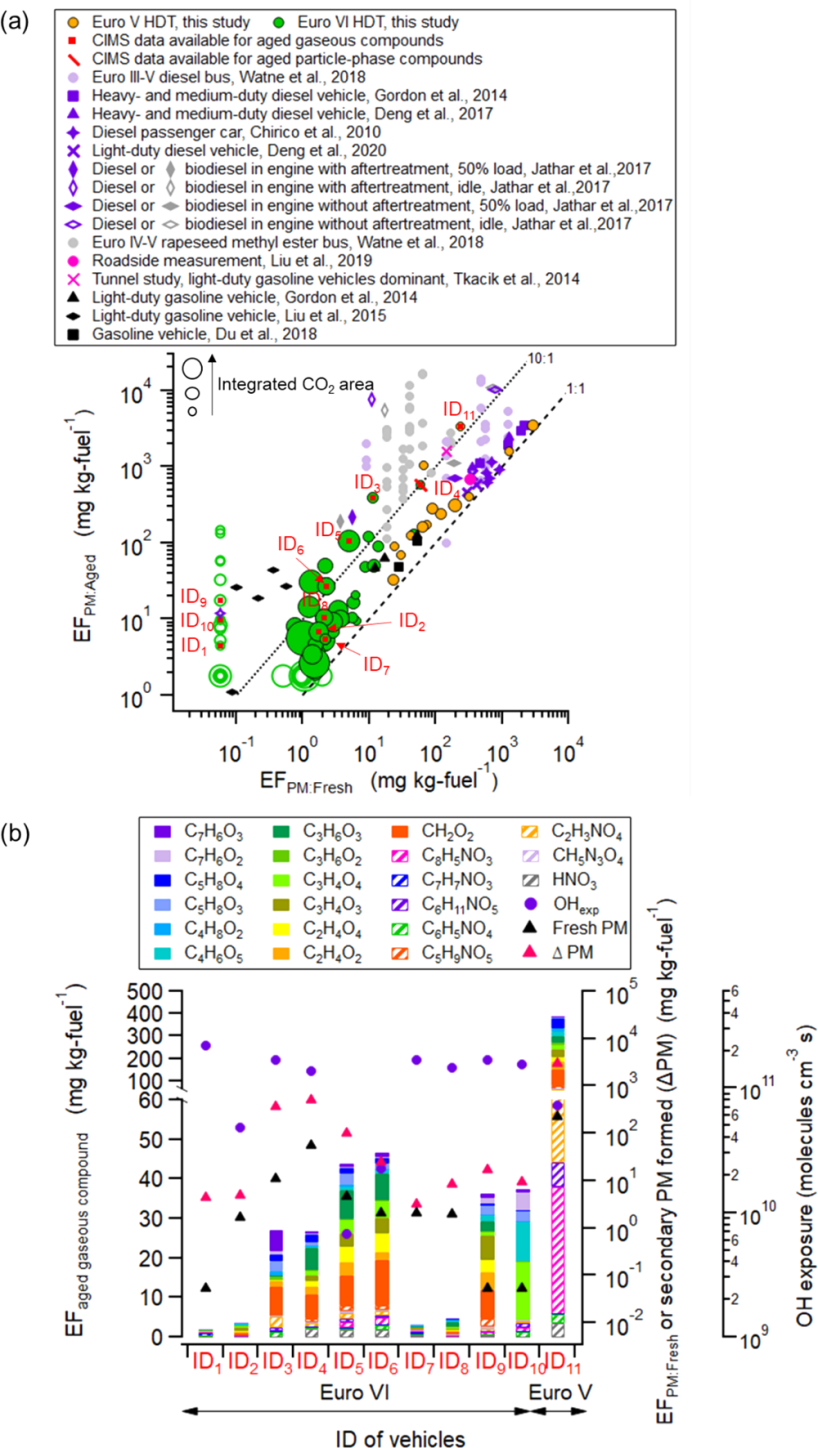
(a) EF_PM:Aged_ vs EF_PM-Fresh_ for Euro V (# =14) and VI (# =80) compliant HDTs determined in this study and comparison with that reported from a tunnel study^18^ and a roadside measurement of a mixed fleet (the median EF),^22^ OFR and chamber studies of older diesel,^17,21,48−51^ biodiesel^21^ and gasoline vehicles.^19,52,53^ Pink, purple, grey, and black symbols represent the roadside study, diesel vehicle, biodiesel vehicle, and gasoline vehicle studies, respectively. The red rectangles and slash represent the HDTs with measurements of aged gaseous- and particle-phase pollutants, respectively, determined by the HR-ToF-CIMS. Open markers indicate the HDTs that had either EF_PM-Fresh_ or EF_PM-Aged_ lower than the detection limit. EF_PM-Aged_ and EF_PM-Fresh_ in this study are presented as a function of the dilution level of the plumes (integrated CO_2_ area, range: 100 to 2 × 10^4^ ppm s). The black dashed lines denote the 1:10 and 1:1 lines. (b) Fresh EF_PM_, aged gaseous emissions and secondary particle mass formed (ΔPM= EF_PM-Aged_ − EF_PM-Fresh_) for individual HDTs at the respective OH_exp_. Table S5 gives the summary of methods used for selected studies. Note that aged gaseous emissions for Euro V-compliant vehicles were derived from one available HDT so are only indicative.

For each of the 94 HDTs, the total particle mass with and without further oxidation was measured using the EEPS (EF_PM-Aged_ and EF_PM-Fresh_, respectively). In Figure 5a, the measured EF_PM-Fresh_ and the corresponding EF_PM-Aged_ for Euro VIs and Euro Vs were compared to those reported from a tunnel study^18^ and a roadside measurement of a mixed fleet,^22^ OFR and chamber studies of older diesel,^17,21,48−51^ and biodiesel^21^ and gasoline vehicles.^19,52,53^ For most of the HDTs studied, the particle mass increased when the emissions were oxidized. Around half of the Euro VIs had either EF_PM-Fresh_ or EF_PM-Aged_ lower than the detection limit (denoted by open circles), indicating that either they were clean emitters with EF_PM-Aged_ lower than around 2 mg kg fuel^–1^ or their primary emissions were too dilute for detection, that is, those with a small CO_2_-integrated area are found to the left in Figure 5a. The average EF_PM-Aged_ of Euro VIs was approximately 30 mg kg fuel^–1^, almost 30 times lower compared to that from Euro Vs. However, the median ratio of EF_PM-Aged_ to EF_PM-Fresh_ was larger for Euro VIs (3.3–12.4) than for Euro Vs (2.4). The range for Euro VIs depends on the exclusion or inclusion of HDTs with EF_PM_ below the detection limit; hence the ratio can be between 3.3 and 12.4. The EF_PM-Aged_ measurements for Euro VIs in this study were considerably lower than those for Euro III–V diesel- or biodiesel-fueled buses^21^ (100 to 1.7 × 10^4^ mg kg fuel^–1^) and heavy-duty or medium-duty diesel vehicles without after-treatment devices^48,49^ (962–3460 mg kg fuel^–1^). The EF_Aged-PM_ of Euro V was comparable to that of diesel (or biodiesel)-fueled engines with and without after-treatment devices under 50% load condition by Jathar et al.^50^ It is noted that idling conditions and cold starts could provide much higher EF_Aged/PM_.^15,50,54^ Interestingly, the EF_PM-Aged_ for Euro VIs was also within the range of values for gasoline vehicles compliant to Euro 5, pre-Euro 5, California LEV 2, and pre-LEV2 standards (1–124 mg kg fuel^–1^).^19,52,53^ It should be noted that the EF_PM-Fresh_ and EF_PM-Aged_ values presented here include both organic and inorganic aerosol masses, while some studies only report the organic fraction. This does not change the conclusion that Euro VIs generate the lowest secondary mass when compared to older diesel vehicles.

The 11 HDTs with the aged gaseous data available, shown with red rectangles in Figure 5a, were analyzed more thoroughly. The secondary particle mass formed (ΔPM = EF_PM-Aged_ – EF_PM-Fresh_) and the aged gaseous emissions for these individual HDTs are shown in Figure 5b. Vehicles ID_1_ to ID_10_ were Euro VIs, while Vehicle ID_11_ was a Euro V. Four of the Euro VIs (ID_1,2,7&8_) had both low-aged gaseous emissions and moderate ΔPM, indicating that they were fairly clean vehicles. The other Euro VIs had substantially higher-aged gaseous emissions with higher and highly variable ΔPM. The secondary PM formed is dependent on several factors such as the mass of emitted precursors, OH_exp_, and the available surface area for the condensational sink. The hydrocarbon precursor emission rates, affected by engine load conditions, influence the amount of secondary gaseous pollutants and PM formed.^10^ However, the secondary production of gaseous pollutants and PM can be driven by specific subsets of hydrocarbon precursors and at different time scales.^10^ For two of the Euro VIs (ID_5&6_), the OH_exp_ was substantially lower compared to the other Euro VIs, which might explain the lower ΔPM formed compared to HDTs ID_3&4_. For HDTs ID_9_ and ID_10_, moderate amounts of aged gaseous compounds were formed but a relatively low secondary PM was produced (compared to ID_3_–ID_6_). Here, the freshly emitted particles, which were significantly lower compared to ID_3_–ID_6_, may not provide enough surface area/volume for an efficient partitioning/condensational sink. Compared with other HDTs, ID_10_ had a slightly different secondary acid profile (dominated by malonic and malic acid), which indicates a different composition of precursors that are less prone to form secondary PM. Consistent with the higher fresh emissions from Euro Vs, ID_11_ (Euro V) produced the highest amount of aged pollutants including ΔPM, significantly higher than all the other Euro VIs. The median ΔPM values for Euro V and Euro VI vehicles were 111 and 3.2 mg kg fuel^–1^, respectively. Thus, a shift to a fleet dominated by Euro VIs would not only result in the reduction in primary pollutant emissions but also be beneficial for mitigating secondary pollutant production including PM and numerous gaseous pollutants.

### 3.4 Atmospheric Implications

Over the years, the European Union has implemented regulations for heavy-duty vehicles to reduce street-level pollutant concentrations, such as introducing stricter emission standards and substituting traditional diesel fuels with biodiesels.^55^ These regulations target the primary emissions of a few species from a vehicle, while non-regulated compounds and secondary pollutants can also have serious negative impacts on air quality and human health. A question that arises is whether the reduction of compounds targeted in current legislation also reduces other pollutants. From our study, it is evident that this is the case for a majority of the measured primary pollutants and is reflected in the reduction of secondary pollutants as well. However, the reduction of the particle phase has mostly been for the non-volatile fraction, while the volatile fraction and the compounds investigated using the FIGAERO-ToF-CIMS have been reduced to a lesser degree by the newer technologies. In addition, the risk with some abatement systems is their potential to generate very specific chemical compounds including the by-products from the urea-SCR exhaust systems such as isocyanic acid and urea nitrate. However, these compounds were reduced significantly for Euro VIs, and it is less likely that these, when a full transition to the modern technology has been implemented, will be of great concern. On the contrary, a concern that remains, evident from the NMF analysis, is the issue of maintenance. This will be relatively more important for air quality in the future when the emission of pollutants produced during normal operation has decreased significantly. Another future concern is that once emitted into air, the emissions will be subjected to atmospheric processing, that is, aging. Previous studies have suggested secondary anthropogenic sources of formic, butyric, and propanoic acids.^10,56,57^ These sources have been confirmed along with the additional formation of malonic, malic, and pyruvic acid. Secondary PM formation resulting from exhaust aging was significant for both Euro Vs and VIs (median EF_PM-Aged_/EF_PM-Fresh_ ratios of 2.4 and 3.3–12.4, respectively). Furthermore, an astonishing overall reduction of 90% in the production of secondary PM for Euro VIs was found, compared to the Euro Vs studied here but also in comparison to the results of previous studies of diesel vehicles.^17,21,48,49^

The effect of a full transition to Euro VI compliancy can be illustrated using the current Swedish fleet. During 2018, the distances driven by Euro V and VI HDTs on Swedish roads were 1.3 × 10^9^ and 2.2 × 10^9^ km, respectively, accounting for 32 and 53% of the total distance driven by Swedish diesel HDTs, respectively. The remaining 15% was attributed to Euro 0–IVs. Based on the average primary EFs (Table 1), the replacement of the current HDT fleet with a fleet consisting exclusively of Euro VIs would reduce the annual primary emissions of non-nitrogen, nitrogen-containing species, and formic acid by 87, 64, and 87%, respectively. However, it will imply only a 10% reduction in the annual emissions of HONO. In comparison, the emissions of typical combustion pollutants were estimated to decrease annually by 94, 93, 74, 18, and 6% for BC, PM, NO_*x*_, CO, and PN, respectively. In addition, the aged annual emissions of gaseous non-nitrogen and nitrogen-containing compounds and PM would yield similar reductions of 84–92% when replacing the fleet. Hence, the adoption of Euro VIs results in a significant reduction in the production of both primary and secondary air pollutants.

## Supporting Information Available

Description of NMF and HCA; EF calculations; model calculations of OH exposure; uncertainty discussion; schematic of the experimental setup; average pollutant EFs; cophenetic correlation coefficients and matrices; HMHC and LMLC factors profiles; carbon number; dendrogram from HCA; EF_NOx_, EF_PM_, and EFs; EF_Fresh_ and EF_Aged_ of PM contributing species; average EFs of gaseous compounds; top 10 compounds for NMF factors; average fresh and aged EFs; and reaction and rate coefficients (PDF)
