# Supplementary material for: Emissions and Secondary Formation of Air Pollutants from Modern Heavy-Duty Trucks in Real-World Traffic—Chemical Characteristics Using On-Line Mass Spectrometry
Source: Environ Sci Technol. 2021 Oct 15;55(21):14515–25. doi: 10.1021/acs.est.1c00412 (PMC8567417; doi:10.1021/acs.est.1c00412)
Supplement: Supplementary file 1 — es1c00412_si_001.pdf [file es1c00412_si_001.pdf]

Supplemental information Zhou ***et al.***

Emissions and secondary formation of air pollutants from modern heavy-duty trucks in real-world traffic – chemical characteristics using on-line mass spectrometry

Liyuan Zhou[1], Christian M. Salvador[2], Michael Priestley[2], Mattias Hallquist[2*], Qianyun Liu[1],Chak K. Chan[1*] and Åsa M. Hallquist[3]

[1]School of Energy and Environment, City University of Hong Kong, Hong Kong, China
[2]Department of Chemistry and Molecular Biology, University of Gothenburg, Gothenburg, Sweden
[3]IVL Swedish Environmental Research Institute, Gothenburg, Sweden

Correspondence to: M. Hallquist (hallq@chem.gu.se) and Chak K. Chan (Chak.K.Chan@cityu.edu.hk)

Number of pages: 22
Number of Figures: 9
Number of Tables: 6

Description of Non-negative matrix factorization (NMF) and Hierarchical cluster analysis (HCA)
Description of emission factor calculations
Description of model calculations of OH exposure
Uncertainty discussion
Figure S1, Schematic of the experimental setup and examples of raw data
Figure S2, Illustration of repeatability for chemical compounds
Figure S3, Cophenetic correlation coefficients
Figure S4, Cophenetic correlation matrices.
Figure S5, HMHC and LMLC factors ion profiles
Figure S6, HMHC and LMLC factor characteristic
Figure S7, Dendrogram from HCA of the vehicles
Figure S8, Cluster characteristics
Figure S9, Emission factors of particle-phase pollutants
Table S1, Average emission factors (EFs)
Table S2, Top 10 compounds for NMF factors
Table S3, Particle-phase emission factors
Table S4, Reactions and rate coefficients for OH_{exp} model calculations
Table S5, Summary of selected studies and methods to derive secondary PM for Figure 5
Table S6. Background variation and its influence on uncertainties on uncertainties of derived EFs

Non-negative matrix factorization (NMF) and Hierarchical cluster analysis (HCA)

NMF

Where the dimensionality of a dataset is large, it is useful to reduce that dimensionality to better understand the properties or processes of the system under study. Non-negative matrix factorization (NMF)[1] is a dimensionality reduction technique used in various fields such as atmospheric chemistry[2, 3] to simplify data interpretation.

Observations represented as a positive matrix V, with dimensions (n, m), are approximated by the factorized matrices W, with dimensions (n, k); and H, with dimensions (k, m); where k is the selected number of factors.

$$\boldsymbol{V} \approx \boldsymbol{WH}$$

Elements of W and H are updated iteratively until a cost function is minimized and the chosen solution reached[2, 4]. The cost function used here is the sum of the squares of the difference between the observation matrix V and its reconstruction, WH (Frobenius norm):

$$d(V, WH) = \frac{1}{2}\sum_{i,j}\left(V_{ij} - WH_{ij}\right)^2$$

W and H are updated iteratively using the following formulae[4] as implemented by the sklearn.decomposition.nmf (0.20.0) package in python 3.6:

$$W_{i,a} = W_{i,a}\,\frac{(VH^T)_{i,a}}{(WHH^T)_{i,a}}; \quad H_{a,j} = H_{a,j}\,\frac{(W^TV)_{a,j}}{(W^TWH)_{a,j}}$$

Here we use NMF to find common factors in CIMS EFs for the sampled vehicles. To find the number of factors for decomposition (k) we use the cophenetic correlation coefficient (CCC) as performed in other studies[2, 5]. Each NMF run is initiated with a random seed 20 times to ensure the chosen solution is not a local minimum and to ensure reproducibility. The optimization is allowed to run for 500 iterations. Once k has been selected, the run with the lowest residual error (distance) is selected as the chosen solution.

HCA

Hierarchical cluster analysis (HCA) is a technique used to group observations into clusters that are more easily interpretable than individual observations. HCA is agglomerative such that individual observations merge into clusters, which can then merge further into larger clusters.

HCA is independent of calibration instead it aims to describe the similarity of the trends of observations, which is useful here where measurements span several orders of magnitude.

Here we use HCA to group vehicles into clusters using their NMF factors derived from the CIMS EFs. The implementation of HCA as used in this instance is described elsewhere[6]. Briefly, the similarity between two observations (A and B) are found by minimizing the root of the sum of squares of a pair of observations (Vehicle EFs, i).

$$d_{A,B} = \sqrt{\sum_{i}(A_i - B_i)^2}$$

The Ward linkage criterion was chosen to determine the distance between sets of observations as it gave similar or more interpretable results compared with other linkage criteria. Where the mean square error between a pair of candidate clusters and their subsequent merge cluster is minimal, the clustering is allowed to proceed; see Priestley et al.[6] for more detail. The user then determines the final number of clusters. This decision is dependent on the level of granularity required to interpret and describe the system. HCA was implemented using the cluster hierarchy module from SciPy (1.2.0) scientific python library using Python 3.6.

Analysis procedure

As many of the vehicles exhibit no detectable EF for a given species, 223 ions that have a data coverage of 75% of the 73 passages are selected for factorization. This data coverage gave the best trade-off between useful data whilst keeping the highest number of dimensions available for NMF. To remove the effect of dominant EFs skewing the process, data were scaled by first taking the log and then scaling between 0 and 1. Any missing values were imputed with the value 0. For the NMF analysis, a range of 2 to 11 factor solutions are explored, which were initialized at 20 randomly seeded points. 500 iterations were allowed before reaching the final solution. The NMF run with the lowest reconstruction error of a given factor solution is chosen as the optimum solution. Here a 2-factor solution is chosen (Figure S3). The cophenetic correlation matrices can be found in Figure S4.

Factor 1 is the dominant factor for all vehicles although magnitudes vary. Contrastingly, factor 2 is the minority factor and can vary in magnitude from 0, i.e. it is not present at all, to nearly 50% of the total factor contribution (Figure 3a). Analysis of the top ten species for each factor show Factor 2 is a low molecular weight factor that contains a strong contribution from small organic and inorganic molecules. Conversely, Factor 1 is a high molecular weight factor

comprising many high mass organic compounds. The average mass for the top ten contributors is 287 ± 62 (1σ) for Factor 1 and 93 ± 33 for Factor 2. This separation of low and high mass factors is demonstrated in Figures S5 and S6. For these reasons, factor 1 is designated high mass and high carbon (HMHC) factor, whereas factor 2 is designated low mass and low carbon (LMLC) factor. These species are listed in Table S2. The 73 passages for which the identity is known were then clustered by HCA using these two NMF derived factors as the input variables. Analysis of the dendrogram suggests 2,3,4,5 and 10 cluster solutions are possible (Figure S7).

Emission factor calculations

The EF calculation was based on the carbon balance method[7, 8], and details have been given in our previous studies and for this specific campaign in the overview paper by Zhou, et al. [9]. EFs were calculated using equation X1:

$$EF_{pollutant} = \frac{\int_{t_1}^{t_2}([pollutant]_t - [pollutant]_{background})dt}{\int_{t_1}^{t_2}([CO_2]_t - [CO_2]_{background})dt} \times EF_{CO_2}, \quad (X1)$$

where $EF_{pollutant}$ is the emission factor of the respective pollutant. The time interval of t_1 to t_2 represents the period when the instruments measured the concentration of an entire pollutant peak from an individual HDT; typical duration of plumes can be seen in Figure S1. t_1 and t_2 were determined independently for each pollutant peak to account for differences in the response time of individual instruments to the exhaust plume. The starting time (t_1) can easily be identified, while t_2 is when the intensity after the peak levels out and becomes indistinguishable from background levels. It is noted that the total integrated peak intensity usually is insensitive to the exact location of t_2 since the added integrated signals at or beyond this point are small and represent the noise around the background level. The background concentrations (except for FIGAERO-particle phase) were derived using data points just prior (ca 5 seconds) to the concentration peak for individual HDTs. This minimizes any effects of fluctuation of ambient concentration. At this specific background site, the general variation in background concentration was low for all pollutants and varied on timescales much longer than the duration of each measurement. To get an emission factor per kg fuel, an EF_{CO2} of 3158 g (kg diesel fuel)$^{-1}$ was used assuming complete combustion and a carbon content of 86.1 % as given in Edwards, et al. [10].

Model calculations of OH exposure

The OH_{exp} in Go:PAM was calculated using the model described by Watne, et al. [11]. Briefly, a chemical model containing a comprehensive description of ozone photolysis and HO_x chemistry and a skeleton description of NO_x, CO, HC and SO_x chemistry was used to mimic the gas-phase chemistry in Go:PAM (Table S4). The minimum OH exposure was derived for each HDT passage plume using the maximum NO_x, HC and CO concentrations in Go:PAM and the corresponding water and ozone concentrations. The assumed speciation of HC was aldehydes (26%), alkanes (33%), alkenes (14%) and aromatic compounds (27%). The oxidation capacity of Go:PAM was offline calibrated by SO_2 as described by Lambe et al.[12], where the photon flux at 254 nm, $P_{FLUX254}$= 1.57×10^{16} cm^{-2} $s^{-1,}$ and first order loss rates of OH were derived by matching the measured and modeled SO_2 and O_3 decreases.

Recently, a concern of non-OH chemistry in the OFR has been raised.[13] In this study, we estimated the ratios of exposures of non-OH species to OH exposure for O_3, $O(^1D)$ and $O(^3P)$, and they were generally on the orders of 10^3, 10^{-7} and 10^{-3} cm s^{-1}, respectively. The relative importance of non-OH chemistry was evaluated according to Peng et al.[13], by taking toluene as a surrogate as it is a common SOA precursor found in vehicle emissions.[14, 15] The undesired VOC destructions by O_3, $O(^1D)$ and $O(^3P)$ were negligible (close to 0 %). The direct photolysis of aromatics in Go:PAM has been evaluated by Watne et al.[11] under similar experimental conditions (photon flux, residence time). No reductions of toluene and trimethyl-benzene were observed with UV light on.

Uncertainty discussion

The uncertainties of derived EFs can be divided into analytical uncertainties for respectively instrument, the combined error on the method to derive EFs (e.g., background correction) and the overall variability of conditions for the on-road vehicle (e.g., engine speed). All these uncertainties are then contributing to the variability of the observed EF of each vehicle class as presented in Table 1 and Table1S.

Analytical uncertainties for respectively instrument

Uncertainties in CO_2 and NO_x measurements were estimated to be around ±2% for the LI-840 analyzer and ±1% for the chemiluminescent analyzers (model 42i, Thermo Scientific Inc.), respectively. For the remote sensing device measurements (AccuScanTM RSD 5000) (OPUS Inspection Inc.) of CO, NO_x, and HC, the uncertainties were about ±15% of the readings[16] (these data was not used for EF calculations but rather to estimate OH exposure for the aged data). For EEPS data the EF_{PN} was compared with EF derived from a CPC unit (see Figure S1 in Zhou et al, 2020). The uncertainty in the slope has a relative standard error of 3%. The uncertainties in the EF_{PM} is more complex to derive and depends on the sizing and nature of emitted particles that for combustion generated particles is somewhat unknown. However, previous diesel engine tests showed that the deviation of the total particle number concentrations measured by the EEPS (soot matrix) and SMPS differed by less than 26%.[17] This corresponds to about 13% in mass concntrations.[17] Consequently similar uncertainties may be assumed for this study. For HR-ToF-CIMS measurements, a sensitivity factor to convert the CIMS signal into concentration is necessary to estimate absolute EFs. The accuracy of the pollutant concentration was limited by the uncertainty in the sensitivity factor. Based on Lopez-Hilfiker et al.[18], the maximum sensitivity (collision-limited) in this study was determined to be 20 Hz ppt^{-1}, which falls within previously reported ranges. Using the maximum sensitivity provides a lower-limit estimate of EF for all the oxygenated volatile organic compounds (OVOCs).[19] One may note that the instrument sensitivity do not change during the campaign and do not vary between captured HDTs and therefore do not influence the conclusions of observed relative emission reductions as a result of the change from Euro V to Euro VI or the ratio of aged to fresh emissions.

Error due to background correction

As described in the emission factor calculations section (SI), the consideration of the background for gas- and particle-phase constituents (except for FIGAERO - particle phase) is straightforward, i.e., an average (ca 5s) of the signal prior to the plumes. The uncertainty in

background correction relied on the potential variation of pollutant background concentrations, which, however, were relatively stable during the 5s-duration before each peak (Figure S1). The relative standard deviation of the background varied for each passage and species with an average of 27% for the 4672 background averages used (73 passages × 64 species matrix). However, the absolute influence on the EF is usually less since the signal is greater than the background. Therefore, the uncertainties arisen from background subtraction were estimated to be generally less than 20% (see examples in Table S6).

Variability within the fleet

The largest influence on the EF derived in this study come from the variability of the fleet and the real-world driving conditions, which were greater than any of the analytical and background correction uncertainties. One may note that this variability is an important aspect of how EFs do vary between vehicles and illustrates real conditions rather than being an uncertainty in the measurements. To analyse the repeatability for the method as such one may consider multiple passages of the same vehicle. However, the current study is limited by the small number of multiple passages of the same vehicle while for the data presented in Zhou et al. [9], the numbers of multiple passages were greater enabling a study on the PN/PM/NO_x emissions variability/reproducibility. Briefly, 55 out of 330 Swedish HDTs passed the sampling location repeatedly and yielded 137 plumes. The average pollutant emission factors of each HDT plotted against the individual plume measurements of the corresponding HDT were presented (Figure S6 in Zhou et al.,[9]). The EEPS measured PM showed little variation (R^2=0.77) among multiple passages of the same HDT. This indicates the credibility of our measurements to capture real-world driving conditions while the overall spread, adding the variation in the fleet, is reflected by the range presented in Table 1.

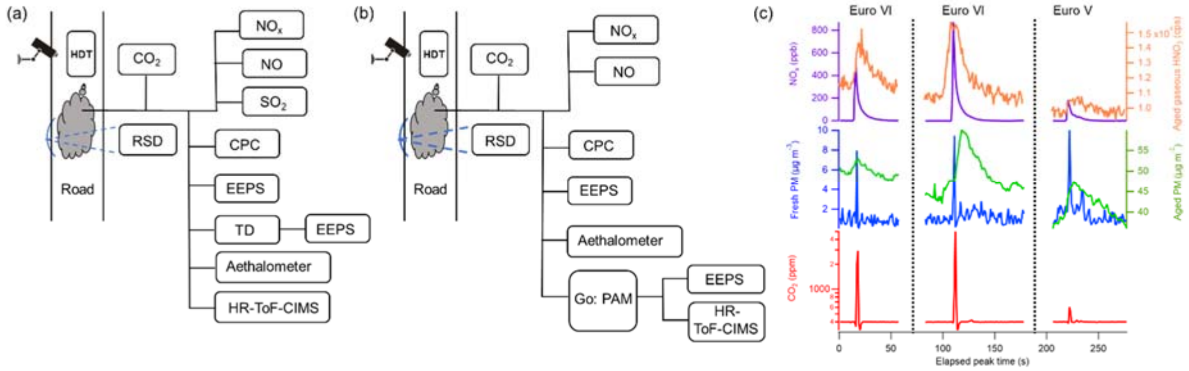

Figure S1. Schematic of the experimental setup for (a) fresh (same as used in Zhou et al.[9]) and (b) aged emission measurements and (c) examples of temporal profiles of pollutant concentrations. For aged setup (panel b, using Go:PAM) measurements, the ambient air always went into the Go:PAM. When UV was on, the samples were aged. The samples were continuously oxidized in the Go: PAM and the outflows were subsequently characterized by the HR-ToF-CIMS. The rise and fall (peaks) in measured pollutant concentrations indicated HDT passages (c) and the oxidized ambient air prior to the concentration peak for individual HDTs was used as the background condition. The time delay between a passage and the signal peak was considered.

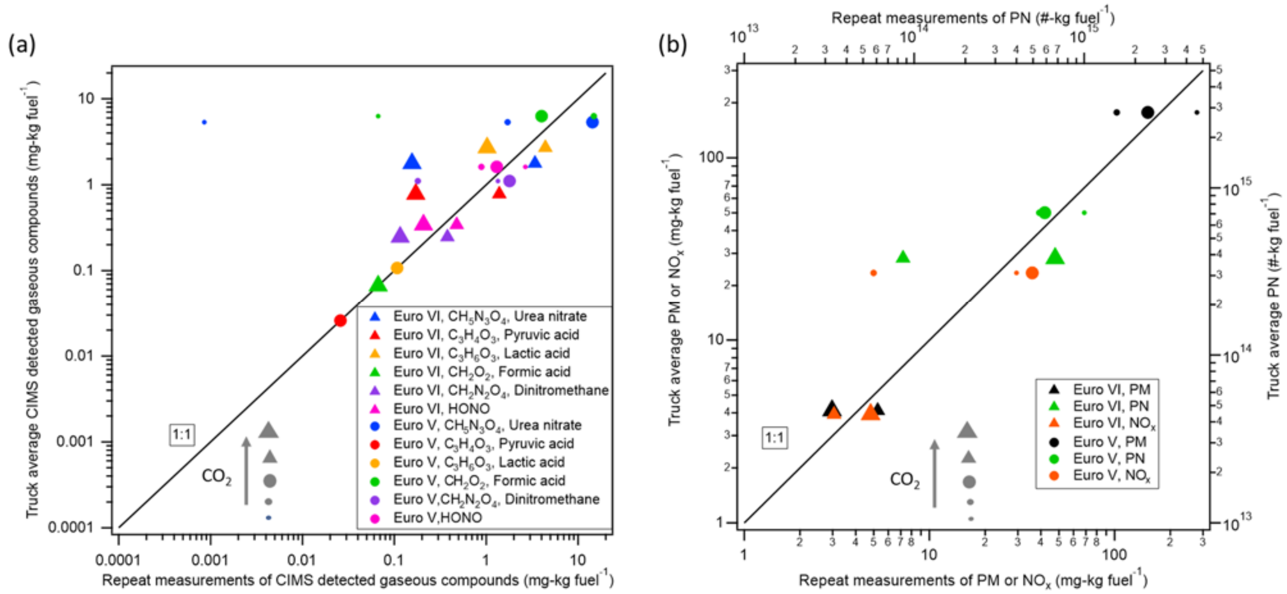

Figure S2. Average pollutant emission factors of (a) CIMS detected gaseous pollutants and (b) PM, PN, and NO_x for each HDT as a function of the individual plume measurements of the corresponding HDT. The size of the symbols represents the degree of dilution (i.e., the integrated CO_2 concentration).

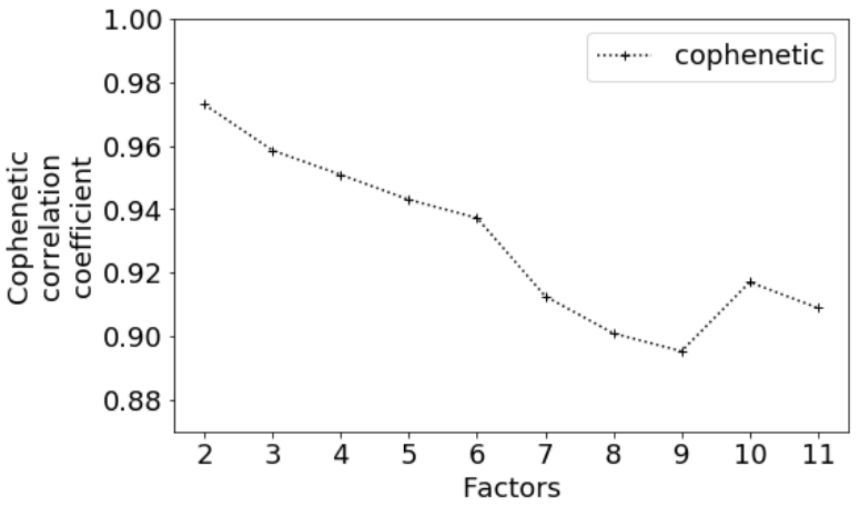

Figure S3. Cophenetic correlation coefficients for the optimum (minimum reconstruction error) for the different factor solutions. The highest value of 0.975 is found for the 2-factor solution.

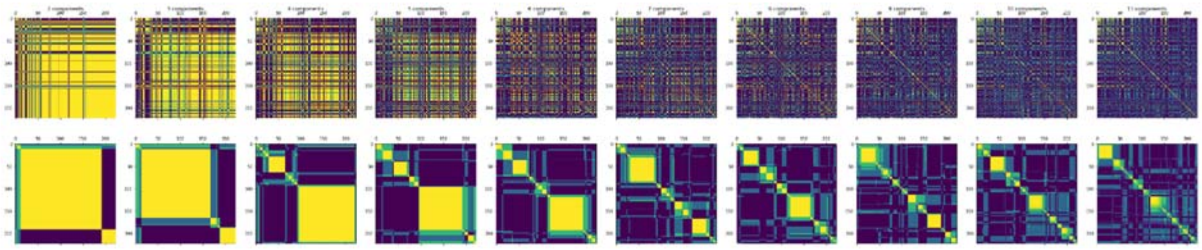

Figure S4. Cophenetic correlation matrices. Columns are the number of factor solutions. The top row is unordered and the bottom row is ordered.

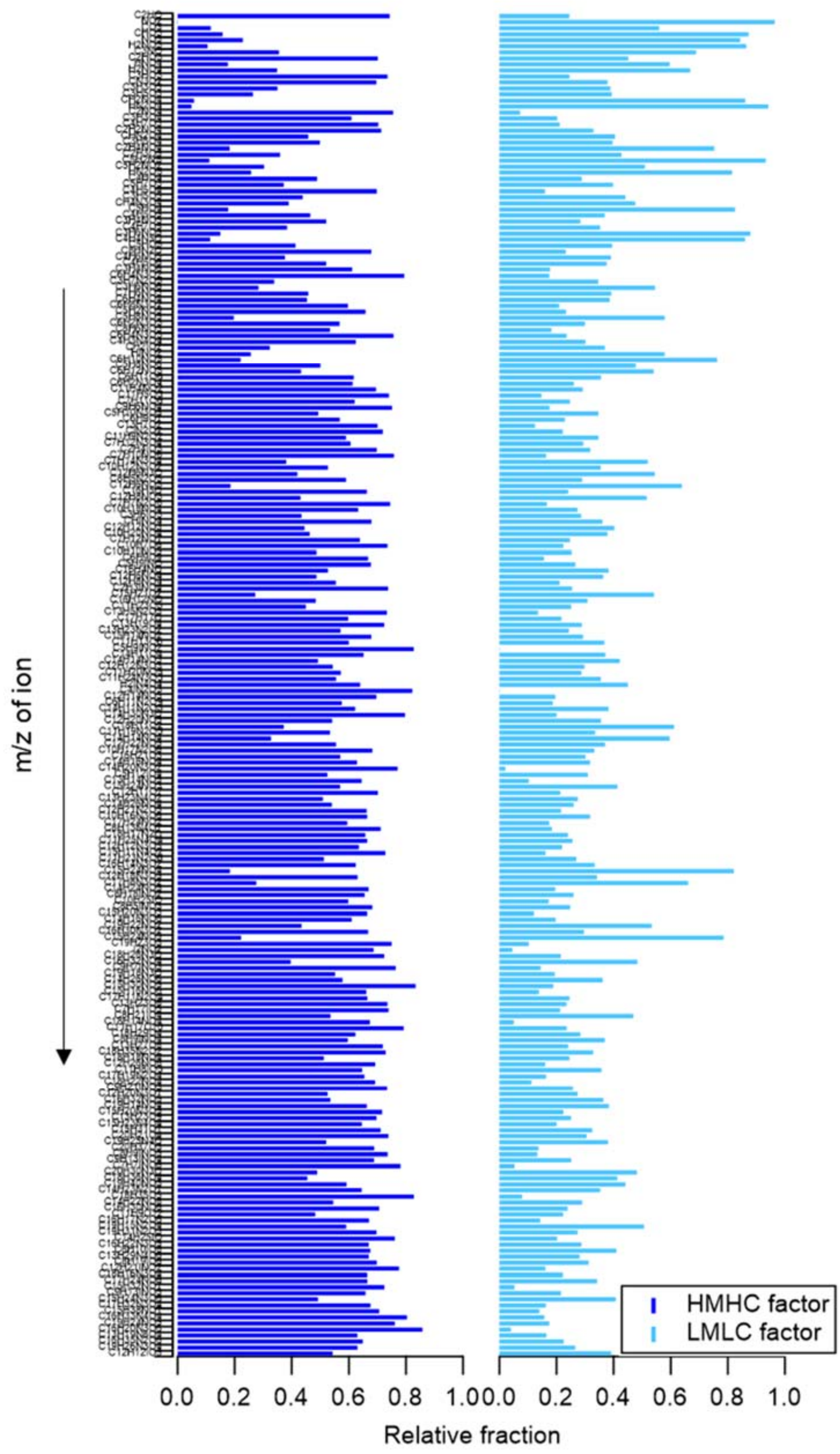

Figure S5. HMHC and LMLC factors profiles.

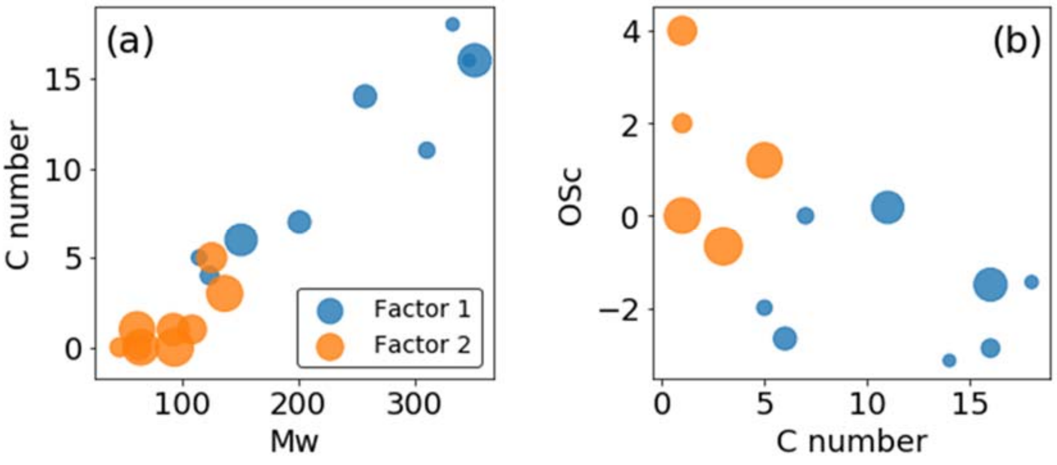

Figure S6. (a) Carbon number as a function of molecular weight for the top 10 ions in two factors. (b) Average carbon oxidation state as a function of carbon number for top 10 ions in two factors, only organic species of factor 1 are shown.

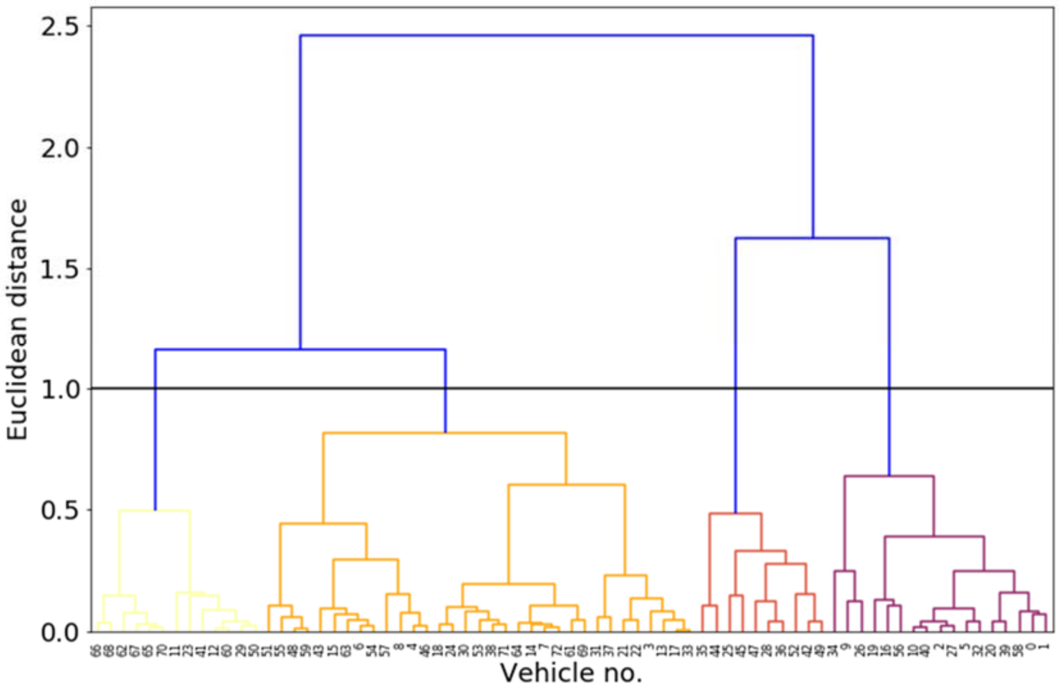

Figure S7. Dendrogram from HCA of the vehicles. A four-cluster solution is chosen.

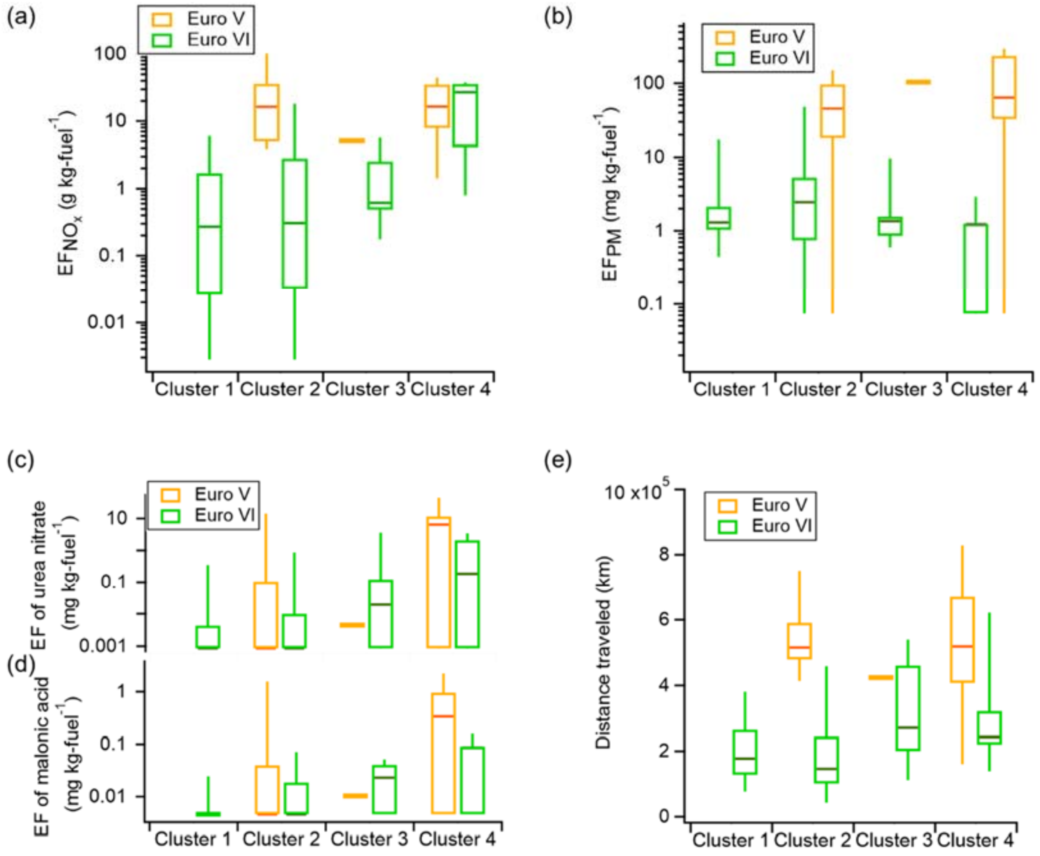

Figure S8. (a) EF_{NOx}, (b) EF_{PM} , EFs of gaseous (c) urea nitrate, (d) malonic acid and (e) distance traveled for Swedish Euro V and Euro VI HDTs. Non-detectable pollutant emission signals for captured plumes have been replaced by the corresponding minimum value among all recorded emission factors. For box-and-whisker plots, the top and the bottom line of the box are 75th and 25th percentiles of the data, the dark orange and green lines are the medians, and the top and bottom whiskers are 90th and 10th percentiles.

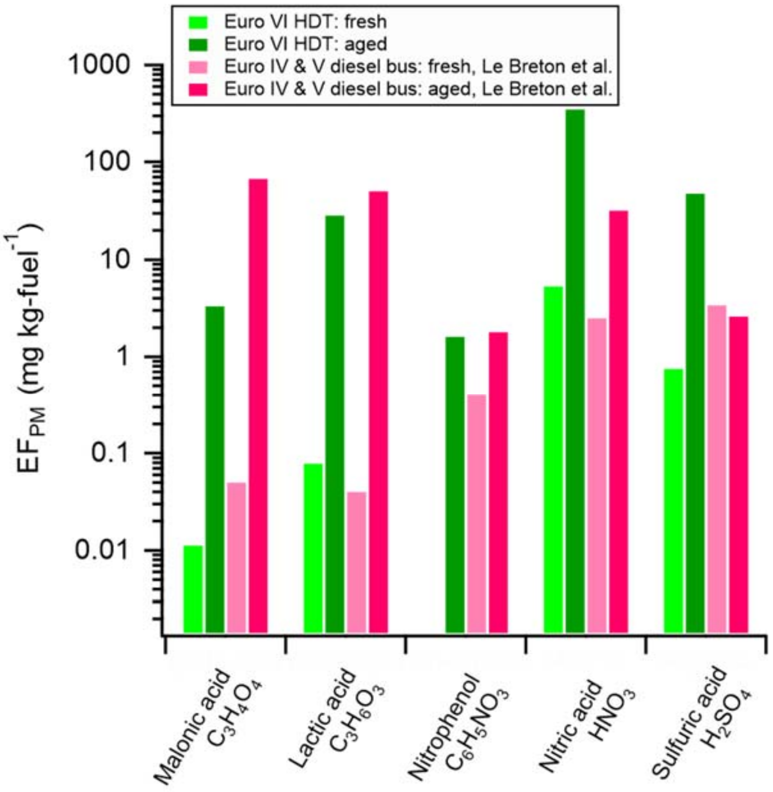

Figure S9. EF_{Fresh} and EF_{Aged} of PM contributing species in Euro VI HDT emissions in this study (EF_{Fresh} - average EFs from two HDTs and EF_{Aged} derived from one HDT) and comparison with Euro IV&V diesel bus emissions[20]. OH_{exp} was 1.4×10^{11} molecules cm^{-3} s.

Table S1. Average emission factors (EFs) of gaseous compounds measured by CIMS for Euro V and VI HDTs. Stated errors are at the statistical 95 % confidence interval. Median EFs are shown in the parentheses.

Formula	Identified as	Fresh EF (mg kg-fuel^{-1})		Reduction of fresh EF from Euro V to VI HDT/%	Aged EF (mg kg-fuel^{-1})		Reduction of aged EF from Euro V to VI HDT/%
		Euro V HDT	Euro VI HDT		Euro V HDT[a]	Euro VI HDT	
			Carboxylic acids				
CH_2O_2	Formic acid	15±12 (4.2)	0.98±0.60 (0.26)	93	74	4.2±3.1 (3.5)	94
$C_3H_6O_3$	Lactic acid	14±10 (1.7)	1.1±0.46 (0.42)	92	28	2.5±2.2 (0.82)	91
$C_2H_4O_4$	Dihydroxyacetic acid	5.5±5.4 (*)	0.23±0.16 (*)	96	19	1.4±1.3 (0.41)	93
$C_2H_4O_2$	Acetic acid	5.4±5.5 (0.20)	0.21±0.17 (0.0053)	96	36	1.4±1.1 (0.91)	96
$C_3H_4O_3$	Pyruvic acid	2.0±1.8 (0.15)	0.14±0.085 (0.022)	93	35	1.6±1.5 (0.61)	95
$C_3H_4O_4$	Malonic acid	0.73±0.47 (0.23)	0.10±0.10 (0.028)	86	20	2.4±3.3 (0.55)	88
$C_4H_6O_5$	Malic acid	0.97±0.77 (0.42)	0.060±0.046 (*)	94	20	1.5±2.2 (0.23)	93
$C_3H_6O_2$	Propionic acid	0.50±0.58 (*)	0.013±0.011 (*)	97	11	0.21±0.20 (0.084)	98
$C_4H_8O_2$	Butyric acid	0.91±1.0 (*)	0.013±0.011 (*)	99	15	0.32±0.34 (0.13)	98
$C_5H_8O_3$	Levulinic acid	3.4±2.5 (0.78)	0.24±0.16 (0.028)	93	41	1.4±0.85 (1.4)	97
$C_5H_8O_4$	Glutaric acid	0.83±0.51 (0.57)	0.046±0.026 (0.009)	94	1.5	0.80±0.51 (0.53)	47
$C_5H_{10}O_2$	Pentanoic acid	0.90±0.79 (*)	0.040±0.037 (*)	96	-	0.29±0.41 (0.002)	-
$C_6H_{12}O_2$	hexanoic acid	1.7±2.0 (*)	0.037±0.038 (*)	98	0.46	0.18±0.21 (0.011)	61
$C_7H_{14}O_2$	Heptanoic Acid	0.88±0.70 (*)	0.032±0.035 (*)	96	-	0.11±0.24 (*)	-
$C_8H_{16}O_2$	Octanoic acid	1.5±1.7 (*)	0.056±0.058 (*)	96	11	0.20±0.17 (0.079)	98
$C_9H_{18}O_2$	Ethylheptanoic acid	2.1±1.9 (0.19)	0.10±0.060 (0.013)	95	-	1.3±1.8 (0.39)	-
$C_{10}H_{20}O_2$	Decanoic acid	1.5±1.6 (*)	0.15±0.12 (*)	90	-	0.18±0.22 (0.085)	-
$C_{12}H_{24}O_2$	Dodecanoic acid	1.0±0.93 (0.02)	0.018±0.017 (*)	98	2.2	0.60±0.80 (0.023)	73
$C_7H_6O_2$	Benzoic acid	2.2±1.6 (0.39)	0.10±0.068 (0.0072)	95	2.4	0.77±0.95 (0.23)	68
$C_7H_6O_3$	Hydroxybenzoic acid	1.1±1.5 (0.016)	0.16±0.14 (0.015)	85	9.3	1.1±1.1 (0.74)	88
			Carbonyl compounds[b]				
C_2H_4O	Acetaldehyde	0.14±0.13 (0.008)	0.013±0.011 (0.007)	91	0.72	0.096±0.14 (*)	87
C_3H_6O	Propanal/Acetone	0.040±0.035 (0.002)	0.004±0.004 (*)	90	0.096	0.12±0.18 (0.021)	-
C_3H_4O	Acrolein	0.073±0.068 (0.027)	0.002±0.002 (*)	97	2.0	0.047±0.039 (0.033)	98
C_4H_6O	Crotonaldehyde/ Methacrolein	0.041±0.035 (0.007)	0.004±0.003 (*)	90	-	0.030±0.064 (*)	-
$C_5H_{10}O$	Pentanal/Pentanone	0.19±0.19 (*)	0.022±0.020 (0.0013)	88	-	0.025±0.023 (0.013)	-
C_7H_6O	Benzaldehyde	0.17±0.18 (*)	0.022±0.022 (*)	87	-	0.010±0.011 (*)	-
C_8H_8O	Tolualdehyde	0.39±0.31 (0.020)	0.033±0.035 (*)	92	2.5	0.073±0.11 (0.0091)	97
$C_{11}H_8O$	Naphthaldehyde	0.30±0.24 (0.031)	0.035±0.037 (*)	88	4.7	0.19±0.37 (*)	96

$C_{15}H_{10}O$	Anthraldehyde	1.7±1.3 (0.22)	0.15±0.14 (0.0025)	91	-	0.34±0.45 (*)	-
$C_3H_4O_2$	Methylglyoxal	0.37±0.30 (*)	0.013±0.012 (*)	96	2.2	0.14±0.14 (0.026)	94
		Nitrogen containing organic and inorganic species					
$C_8H_5NO_3$	Hydroxyphthalimide	0.40±0.30 (0.036)	0.038±0.026 (*)	91	32	0.56±0.54 (0.13)	98
$C_8H_5NO_6$	Nitrophthalic acid	0.10±0.12 (0.0059)	0.008±0.006 (0.0009)	92	0.47	0.21±0.31 (0.0092)	55
$CH_5N_3O_4$	Urea nitrate	8.2±5.9 (5.6)	0.40±0.25 (0.024)	95	0.74	0.25±0.19 (0.19)	66
HNCO	Isocyanic acid	1.3±1.2 (0.061)	0.032±0.029 (0.008)	98	0.94	0.41±0.30 (0.22)	56
$C_6H_5NO_3$	Nitrophenol	0.29±0.24 (0.018)	0.056±0.043 (0.0016)	81	-	0.44±0.62 (0.0083)	-
$C_8H_9NO_3$	Dimethylnitrophenol	0.77±0.86 (*)	0.063±0.089 (*)	92	-	0.16±0.19 (0.019)	-
$C_6H_5NO_4$	Dihydroxynitrobenzene	2.1±1.4 (0.98)	0.13±0.13 (0.022)	94	2.2	0.44±0.38 (0.17)	80
$C_7H_7NO_3$	Nitrobenzyl alcohol	2.7±2.7 (*)	0.15±0.15 (*)	94	0.8	0.21±0.18 (0.047)	-
$C_2H_3NO_4$	Nitroacetic acid	0.22±0.16 (0.082)	0.087±0.061 (0.0098)	60	22	0.78±0.61 (0.74)	96
$C_3H_5NO_5$	Nitrooxypropanoic acid	1.3±0.85 (0.59)	0.23±0.19 (0.032)	82	4.8	0.42±0.41 (0.084)	91
$C_4H_7NO_5$	Nitrooxybutanoic acid	1.6±0.76 (1.2)	0.22±0.18 (0.063)	86	3.7	0.75±0.90 (0.24)	80
$C_5H_9NO_5$	Nitrooxypentanoic acid	5.0±3.8 (1.5)	0.79±0.93 (0.094)	84	13	0.56±0.50 (0.076)	96
$C_6H_{11}NO_5$	Nitrooxyhexanoic acid	5.0±3.1 (3.3)	0.91±1.1 (0.080)	82	6.1	0.35±0.33 (0.0082)	94
HONO	Nitrous acid	5.1±4.2 (2.8)	4.1±4.5 (0.25)	20	3.4	0.17±0.21 (0.0099)	95
HNO_3	Nitric acid	1.1±0.72 (0.40)	0.23±0.20 (0.0038)	79	3.5	0.78±0.66 (0.44)	78
Non-nitrogen containing species (carboxylic acids and carbonyl compounds)		65±56 (9.2)	4.1±2.7 (0.82)	94	338	24±24 (11)	93
Nitrogen containing organic and inorganic species		35±26 (16)	7.4±7.9 (0.59)	79	94	7.5±8.3 (2.4)	92

[a] EFs derived from one Euro V HDT.

[b] Sensitivity for monoaldehydes or monoketones was underestimated, EFs only represent the lower limit.

*Below detection limits.

Table S2. Top 10 compounds for NMF factors.

No.	Factor 1	Factor 2
1	$C_{16}H_{20}N_3O_6$-	NO_2-
2	$C_{15}H_{30}NO_5$-	HN_2O_4-
3	$C_{18}H_{35}O_5$-	CH_2NO_5-
4	$C_5H_9NO_2I$-	$C_3H_6NO_5$-
5	$C_4N_2O_3I$-	CHO_3-
6	$C_{16}H_{33}N_4O_4$-	H_2NO_3-
7	$C_{14}H_{30}N_3O$-	CH_2NO_4-
8	$C_6H_4N_3O_2$-	$C_4H_4N_5O$-
9	$C_{11}H_{17}O_{10}$-	NO_3-
10	$C_7H_7NO_6I$-	HN_2O_6-

Table S3. Average fresh and aged emission factors (EFs) of CIMS detected particle-phase compounds and total PM measured by EEPS of a Euro V and VI HDTs.

Formula	Identified as	Fresh EF (mg kg-fuel^{-1})		Reduction of fresh EF from Euro V to VI HDT/%	Aged EF (mg kg-fuel^{-1})
		Euro V HDT[a]	Euro VI HDT[a,b]		Euro VI HDT[a,c]
$C_3H_6O_3$	Lactic acid	0.19	0.078±0.064	59	28.5
$C_3H_4O_4$	Malonic acid	0.058	0.011±0.013	81	3.3
$C_5H_8O_4$	Glutaric acid	0.063	-	-	4.6
$C_7H_6O_2$	Benzoic acid	0.033	-	-	2.0
$CH_5N_3O_4$	Urea nitrate	0.053	0.011±0.008	79	0.93
HNCO	Isocyanic acid	0.029	-	-	2.4
$C_6H_5NO_3$	Nitrophenol	0.040	-	-	1.6
HNO_3	Nitric acid	12.2	5.3±3.1	56	349
H_2SO_4	Sulfuric acid	-	0.75±0.86	-	47
Total CIMS		12.7	6.2±4.1	51	440
Total PM measured by EEPS		151	9.2±7.8	94	570
% CIMS		8.4	67	-	77

[a] Fresh EFs derived from one Euro V HDT and two Euro VI HDTs, and aged EF derived from one Euro VI HDT.
[b] Stated errors are standard deviations.
[c] EF_{Aged} after the emissions were exposed to OH of 1.0×10^{11} molecules cm^{-3} s.

Table S4. Reactions and rate coefficients for model calculations of OH exposure. The data were taken from the literature as described in Watne et al. * 74% of measured HC, **26% of measured HC

No.	Reaction	k (cm^3 $molecule^{-1}$ s^{-1})
1	O_3+hv → O_2+O(^{1}D)	0.18
2	O(^{1}D)+H_2O → OH+OH	1.99×10^{-10}
3	O(^{1}D)+O_2 → O(^{3}p)+O_2	3.97×10^{-11}
4	O(^{1}D)+O_3 → O_2+O(^{3}p)+ O(^{3}p)	1.2×10^{-10}
5	O(^{1}D)+O_3 → O_2+ O_2	1.2×10^{-10}
6	O(^{1}D)+N_2 → O(^{3}p)+N_2	3.11×10^{-11}
7	O(^{3}p)+O_2+M → O_3+M	6.1×10^{-34}
8	O(^{3}p)+O_3 → O_2+O_2	7.96×10^{-15}
9	O(^{3}p)+OH → H+O_2	3.29×10^{-11}
10	H+O_2 → HO_2	9.57×10^{-13}
11	H+HO_2 → OH+OH	7.2×10^{-11}
12	H+HO_2 → O(^{3}p)+H_2O	1.6×10^{-12}
13	H+HO_2 → H_2+O_2	6.9×10^{-12}
14	OH+OH → H_2O+O(^{3}p)	1.8×10^{-12}
15	OH+OH → H_2O_2	6.29×10^{-12}
16	OH+O_3 → HO_2+O_2	7.25×10^{-14}
17*	HO_2+ HO_2 → H_2O_2+O_2	3.28×10^{-12}
18**	HC+OH →0.7RO_2+0.3HO_2	1.0×10^{-11}
19	HCHO+OH → H_2O+CO+ HO_2	9.2×10^{-12}
20	SO_2+OH → $OHSO_2$	9.59×10^{-13}
21	$OHSO_2$+O_2 → SO_3+HO_2	4.3×10^{-13}
22	NO+O(^{3}p) → NO_2	1.66×10^{-12}
23	NO_2+hv → NO+O(^{3}p)	1.64×10^{-4}
24	NO_2+OH →HNO_3	1.06×10^{-11}
25	NO_2+OH →HOONO	1.79×10^{-12}
26	HO_2+NO → NO_2+OH	8.16×10^{-12}
27	RO_2+NO → RO+ NO_2	9×10^{-12}
28	O(^{1}D)+N_2+M → N_2O+M	2.82×10^{-36}
29	N_2O+O(^{1}D) → N_2+O_2	5.09×10^{-11}
30	N_2O+O(^{1}D) → NO+NO	7.64×10^{-11}
31	O(^{3}p)+HO_2 → OH+O_2	5.87×10^{-11}
32	O(^{3}p)+ H_2O_2 → OH+HO_2	1.7×10^{-15}
33	H+O_3 → OH+O_2	2.89×10^{-11}
34	HO_2+O_3 → OH+O_2+O_2	1.93×10^{-15}
35	HO_2+ OH → H_2O +O_2	1.11×10^{-10}
36	H_2O_2+hv → OH+OH	1.05×10^{-3}
37	HO_2+hv → OH+O(^{1}D)	4.07×10^{-3}
38	OH+ H_2O_2 → HO_2+H_2O	1.8×10^{-12}
39	NO+O_3→ NO_2+O_2	1.95×10^{-14}
40	O(^{1}D)+H_2 → OH+H	1.2×10^{-10}
41	OH+H_2 → H_2O+H	6.67×10^{-15}
42	NO_2+O(^{3}p) → NO+O_2	1.03×10^{-11}
43	NO_2+O(^{3}p) → NO_3	1.61×10^{-12}
44	H+NO_2 → NO+OH	1.28×10^{-10}
45	NO+NO_3 → NO_2+NO_2	2.65×10^{-11}
46	NO_2+O_3 → NO_3+O_2	3.2×10^{-17}
47	CO+OH → CO_2+H	2.4×10^{-13}
48	OH deposition/loss	35
49	CH_3O → HCHO+ HO_2	9.92×10^{3}
50	CH_3OH+OH → HO_2+HCHO	8.95×10^{-13}
51	OH+CH_3OOH → HCHO+OH	4.01×10^{-12}
52	OH+CH_3OOH → CH_3O_2	6.02×10^{-12}
53	CH_3O_2+CH_3O_2 → CH_3OH+HCHO	4.43×10^{-13}
54	CH_3O_2+CH_3O_2 → CH_3O+CH_3O	2.58×10^{-13}
55	CH_3O_2+NO_2 → $CH_3O_2NO_2$	5.88×10^{-12}
56	$CH_3O_2NO_2$ → CH_3O_2+NO_2	1.50
57	OH+CH_4 → CH_3O_2	6.37×10^{-15}
58	CH_3O_2+HO_2 → CH_3OOH	4.74×10^{-12}
59	CH_3O_2+HO_2 → HCHO	4.67×10^{-13}
60	CH_3O_2+NO → CH_3O+NO_2	7.69×10^{-12}

Table S5. Summary of selected studies and methods to derive secondary PM for Figure 5. Instrumentation, the characterized constituents and the density assumption.

Vehicle type	Literature source	Instrument	Constituents	Applied density (g cm^{-3})
Euro VI HDT	this study	EEPS[a]	Total PM	1
Euro V HDT	this study	EEPS[a]	Total PM	1
Euro III-V diesel bus	Watne et al.,[11]	EEPS[a]	Total PM	1
Heavy- and medium-duty diesel vehicles	Gordon, et al. [21]	SMPS[b], AMS[c] and Aethalometer	OA[e] and BC[f]	1
Heavy- and medium-duty diesel vehicles	Deng, et al. [22]	SMPS[b], AMS[c] and Aethalometer	OA[e] and BC[f]	1
Diesel passenger car	Chirico, et al. [23]	AMS[c] and Aethalometer	OA[e] and BC[f]	N.A.[i]
Light-duty diesel vehicle	Deng, et al. [24]	SMPS[b], AMS[c] and Aethalometer	OA[e] and BC[f]	1
Diesel engine	Jathar, et al. [25]	AMS[c], photoacoustic extinctiometer	OA[e] and BC[f]	N.A. [i]
Euro IV-V rapeseed methyl ester bus	Watne et al.,[11]	EEPS	Total PM	1
Roadside measurement	Liu et al.,[15]	SMPS[b], AMS[c] and Aethalometer	OA[e] and BC[f]	weighted by major chemical constituents
Tunnel study, light-duty gasoline vehicles dominant	Tkacik, et al. [26]	SMPS[b], ACSM[d], Aethalometer	OA[e], IA[g] and BC[f]	weighted by major chemical constituents
Light-duty gasoline vehicle	Gordon, et al. [27]	SMPS[b], AMS[c] and Aethalometer	OA[e] and BC[f]	1
Light-duty gasoline vehicle	Liu, et al. [28]	SMPS[b] and AMS[c]	OA[e]	1.4
Gasoline vehicle	Du, et al. [29]	AMS[c], OC/EC[h] analyzer	OA[e] and EC[h]	N.A. [i]

[a] Engine Exhaust Particle Sizer. [b]Scanning Mobility Particle Sizer. [c]Aerosol Mass Spectrometer. [d]Aerosol Chemical Speciation Monitor. [e]Organic aerosol. [f]Black carbon. [g]Inorganic aerosol. [h]Elemental carbon. [i] Not available.

Table S6. Background variation and its influence on uncertainties of derived EFs. For each of the plumes (#=73) one may estimate how the standard deviation of the background measurements (SD BKG) for each of the species (#=64) would give an uncertainty for each of the 4672 EFs, i.e., depending on the signal after BKG correction and the duration of the plume (t_2-t_1). Listed examples are based on data from the plume/species shown in Figure S1c, i.e., for each specie the values are given for the three plumes (left to right).

Species	signal (after BKG correction)[a]	SD BKG × (t_2-t_1)[b]	The fraction of SD BKG × t_2-t_1 to signal
CO_2	3923 /7546 /302 (ppm s)	22 /4 / 17 (ppm s)	0.5% / 0% / 6%
NO_x	2382 / 3920 / 1124 (ppb s)	17 / 6 / 8 (ppb s)	0.7% / 0.1% / 0.7%
Fresh PM	6 / 11 / 14 (μg m^{-3} s)	1 / 1 / 2 (μg m^{-3} s)	21% / 10% / 15%
Aged PM	39 / 485 / 298 (μg m^{-3} s)	8 / 39 / 34 (μg m^{-3} s)	19% / 8% / 11%
Aged gaseous HNO_3	4.1×10^4/9.0×10^4/1.8×10^4 (counts s)	7.4×10^3/8.3×10^3/3.8×10^3 (counts s)	18% / 9% / 21%

[a] $\int_{t_1}^{t_2}([\textbf{pollutant}]_t - [\textbf{pollutant}]_{\textbf{background}})\textbf{dt}$, which is the numerator in the equation X1 in SI.

[b] The time interval of t_1 to t_2 represents the duration of the specific plume.

References

1. Lee, D. D.; Seung, H. S., Learning the parts of objects by non-negative matrix factorization. *Nature* **1999,** *401*, (6755), 788-791.
2. Isokääntä, S.; Kari, E.; Buchholz, A.; Hao, L.; Schobesberger, S.; Virtanen, A.; Mikkonen, S., Comparison of dimension reduction techniques in the analysis of mass spectrometry data. *Atmospheric Measurement Techniques* **2020,** *13*, (6), 2995-3022.
3. Liang, J.; Fairley, D., Validation of an efficient non-negative matrix factorization method and its preliminary application in Central California. *Atmospheric environment* **2006,** *40*, (11), 1991-2001.
4. Lee, D. D.; Seung, H. S. In *Algorithms for non-negative matrix factorization*, Advances in neural information processing systems, 2001; 2001; pp 556-562.
5. Brunet, J.-P.; Tamayo, P.; Golub, T. R.; Mesirov, J. P., Metagenes and molecular pattern discovery using matrix factorization. *Proceedings of the national academy of sciences* **2004,** *101*, (12), 4164-4169.
6. Priestley, M.; Bannan, T. J.; Le Breton, M.; Worrall, S. D.; Kang, S.; Pullinen, I.; Schmitt, S.; Tillmann, R.; Kleist, E.; Zhao, D., Chemical characterisation of benzene oxidation products under high and low NO x conditions using chemical ionisation mass spectrometry. *Atmospheric Chemistry and Physics Discussions* **2020**, 1-24.
7. Ban-Weiss, G. A.; Lunden, M. M.; Kirchstetter, T. W.; Harley, R. A., Measurement of black carbon and particle number emission factors from individual heavy-duty trucks. *Environ Sci Technol* **2009,** *43*, (5), 1419-24.
8. Hak, C. S.; Hallquist, M.; Ljungstrom, E.; Svane, M.; Pettersson, J. B. C., A new approach to in-situ determination of roadside particle emission factors of individual vehicles under conventional driving conditions. *Atmos. Environ.* **2009,** *43*, (15), 2481-2488.
9. Zhou, L.; Hallquist, Å. M.; Hallquist, M.; Salvador, C. M.; Gaita, S. M.; Sjödin, Å.; Jerksjö, M.; Salberg, H.; Wängberg, I.; Mellqvist, J., A transition of atmospheric emissions of particles and gases from on-road heavy-duty trucks. *Atmospheric Chemistry & Physics* **2020,** *20*, (3).
10. Edwards, R.; Larivé, J.; Rickeard, D.; Weindorf, W., Well-to-Wheels analysis of future automotive fuels and powertrains in the European context: Well-to-Tank Appendix 2-Version 4a. *Joint Research Centre of the European Commission, EUCAR, and CONCAWE* **2014**, 1-133.
11. Watne, A. K.; Psichoudaki, M.; Ljungstrom, E.; Le Breton, M.; Hallquist, M.; Jerksjo, M.; Fallgren, H.; Jutterstrom, S.; Hallquist, A. M., Fresh and Oxidized Emissions from In-Use Transit Buses Running on Diesel, Biodiesel, and CNG. *Environ Sci Technol* **2018,** *52*, (14), 7720-7728.
12. Lambe, A. T.; Ahern, A. T.; Williams, L. R.; Slowik, J. G.; Wong, J. P. S.; Abbatt, J. P. D.; Brune, W. H.; Ng, N. L.; Wright, J. P.; Croasdale, D. R.; Worsnop, D. R.; Davidovits, P.; Onasch, T. B., Characterization of aerosol photooxidation flow reactors: heterogeneous oxidation, secondary organic aerosol formation and cloud condensation nuclei activity measurements. *Atmos. Meas. Tech.* **2011,** *4*, (3), 445-461.
13. Peng, Z.; Day, D. A.; Ortega, A. M.; Palm, B. B.; Hu, W.; Stark, H.; Li, R.; Tsigaridis, K.; Brune, W. H.; Jimenez, J. L., Non-OH chemistry in oxidation flow reactors for the study of atmospheric chemistry systematically examined by modeling. **2016**.
14. Gentner, D. R.; Jathar, S. H.; Gordon, T. D.; Bahreini, R.; Day, D. A.; El Haddad, I.; Hayes, P. L.; Pieber, S. M.; Platt, S. M.; de Gouw, J.; Goldstein, A. H.; Harley, R. A.; Jimenez, J. L.; Prevot, A. S.; Robinson, A. L., Review of Urban Secondary Organic Aerosol Formation from Gasoline and Diesel Motor Vehicle Emissions. *Environ Sci Technol* **2017,** *51*, (3), 1074-1093.
15. Liu, T.; Zhou, L.; Liu, Q.; Lee, B. P.; Yao, D.; Lu, H.; Lyu, X.; Guo, H.; Chan, C. K., Secondary organic aerosol formation from urban roadside air in Hong Kong. *Environmental science & technology* **2019,** *53*, (6), 3001-3009.
16. Huang, Y.; Organ, B.; Zhou, J. L.; Surawski, N. C.; Hong, G.; Chan, E. F.; Yam, Y. S., Remote sensing of on-road vehicle emissions: Mechanism, applications and a case study from Hong Kong. *Atmospheric Environment* **2018,** *182*, 58-74.
17. Xue, J.; Li, Y.; Wang, X.; Durbin, T. D.; Johnson, K. C.; Karavalakis, G.; Asa-Awuku, A.; Villela, M.; Quiros, D.; Hu, S., Comparison of vehicle exhaust particle size distributions measured by SMPS and EEPS during steady-state conditions. *Aerosol Science and Technology* **2015,** *49*, (10), 984-996.

18. Lopez-Hilfiker, F. D.; Iyer, S.; Mohr, C.; Lee, B. H.; D'Ambro, E. L.; Kurtén, T.; Thornton, J. A., Constraining the sensitivity of iodide adduct chemical ionization mass spectrometry to multifunctional organic molecules using the collision limit and thermodynamic stability of iodide ion adducts. *Atmos. Meas. Tech.* **2016,** *9*, (4), 1505-1512.
19. Chen, Y.; Takeuchi, M.; Nah, T.; Xu, L.; Canagaratna, M. R.; Stark, H.; Baumann, K.; Canonaco, F.; Prévôt, A. S.; Huey, L. G., Chemical characterization of secondary organic aerosol at a rural site in the southeastern US: insights from simultaneous high-resolution time-of-flight aerosol mass spectrometer (HR-ToF-AMS) and FIGAERO chemical ionization mass spectrometer (CIMS) measurements. *Atmospheric Chemistry and Physics* **2020,** *20*, (14), 8421-8440.
20. Le Breton, M.; Psichoudaki, M.; Hallquist, M.; Watne, Å.; Lutz, A.; Hallquist, Å., Application of a FIGAERO ToF CIMS for on-line characterization of real-world fresh and aged particle emissions from buses. *Aerosol Science and Technology* **2019,** *53*, (3), 244-259.
21. Gordon, T.; Presto, A.; Nguyen, N.; Robertson, W.; Na, K.; Sahay, K.; Zhang, M.; Maddox, C.; Rieger, P.; Chattopadhyay, S., Secondary organic aerosol production from diesel vehicle exhaust: impact of aftertreatment, fuel chemistry and driving cycle. *Atmospheric Chemistry & Physics* **2014,** *14*, (9).
22. Deng, W.; Hu, Q.; Liu, T.; Wang, X.; Zhang, Y.; Song, W.; Sun, Y.; Bi, X.; Yu, J.; Yang, W., Primary particulate emissions and secondary organic aerosol (SOA) formation from idling diesel vehicle exhaust in China. *Science of The Total Environment* **2017,** *593*, 462-469.
23. Chirico, R.; DeCarlo, P.; Heringa, M.; Tritscher, T.; Richter, R.; Prévôt, A.; Dommen, J.; Weingartner, E.; Wehrle, G.; Gysel, M., Impact of aftertreatment devices on primary emissions and secondary organic aerosol formation potential from in-use diesel vehicles: results from smog chamber experiments. *Atmospheric Chemistry and Physics* **2010,** *10*, (23), 11545-11563.
24. Deng, W.; Fang, Z.; Wang, Z.; Zhu, M.; Zhang, Y.; Tang, M.; Song, W.; Lowther, S.; Huang, Z.; Jones, K., Primary emissions and secondary organic aerosol formation from in-use diesel vehicle exhaust: Comparison between idling and cruise mode. *Science of The Total Environment* **2020,** *699*, 134357.
25. Jathar, S. H.; Friedman, B.; Galang, A. A.; Link, M. F.; Brophy, P.; Volckens, J.; Eluri, S.; Farmer, D. K., Linking load, fuel, and emission controls to photochemical production of secondary organic aerosol from a diesel engine. *Environmental science & technology* **2017,** *51*, (3), 1377-1386.
26. Tkacik, D. S.; Lambe, A. T.; Jathar, S.; Li, X.; Presto, A. A.; Zhao, Y.; Blake, D.; Meinardi, S.; Jayne, J. T.; Croteau, P. L., Secondary organic aerosol formation from in-use motor vehicle emissions using a potential aerosol mass reactor. *Environmental science & technology* **2014,** *48*, (19), 11235-11242.
27. Gordon, T.; Presto, A.; May, A.; Nguyen, N.; Lipsky, E.; Donahue, N.; Gutierrez, A.; Zhang, M.; Maddox, C.; Rieger, P., Secondary organic aerosol formation exceeds primary particulate matter emissions for light-duty gasoline vehicles. *Atmospheric Chemistry & Physics* **2014,** *14*, (9).
28. Liu, T.; Wang, X.; Deng, W.; Hu, Q.; Ding, X.; Zhang, Y.; He, Q.; Zhang, Z.; Lü, S.; Bi, X., Secondary organic aerosol formation from photochemical aging of light-duty gasoline vehicle exhausts in a smog chamber. *Atmospheric Chemistry & Physics* **2015,** *15*, (15).
29. Du, Z.; Hu, M.; Peng, J.; Zhang, W.; Zheng, J.; Gu, F.; Qin, Y.; Yang, Y.; Li, M.; Wu, Y., Comparison of primary aerosol emission and secondary aerosol formation from gasoline direct injection and port fuel injection vehicles. *Atmospheric Chemistry and Physics* **2018,** *18*, (12), 9011-9023.
